# Identification and characterization of a broadly neutralizing and protective nanobody against the HA1 domain of H5 avian influenza virus hemagglutinin

**DOI:** 10.1128/jvi.02090-24

**Published:** 2025-04-07

**Authors:** Siqi Xu, Yutong Liu, Chenying Luo, Mengruo Zhou, Ke Wang, Qianmei Xie, Qi Zhang, Qinying Zhang, Qianyu Li, Zhichao Pan, Saixiang Feng, Ming Liao

**Affiliations:** 1 College of Veterinary Medicine, South China Agricultural University 554665, Guangzhou, China; 2 College of Life Sciences, South China Agricultural University 98444, Guangzhou, China; 3 Institute of Animal Health, Guangdong Academy of Agricultural Sciences 117866, Guangzhou, China; 4 Zhongkai University of Agriculture and Engineering 47894, Guangzhou, China; University Medical Center Freiburg, Freiburg, Germany

**Keywords:** influenza virus, yeast two‐hybrid, nanobody, broadly neutralizing, surface plasmon resonance, inhibition mechanism, prophylactic, therapeutic, epitope mapping

## Abstract

The highly pathogenic avian influenza viruses (HPAIVs) of subtype H5, particularly those of the currently circulating clades 2.3.2.1 and 2.3.4.4, are largely responsible for the sporadic human infections that frequently present with a high case fatality rate. Consequently, there is an urgent necessity for the development of advanced antiviral therapeutic options against the H5 HPAIVs. Herein, the yeast two-hybrid system was employed for identifying seven nanobodies that bind the HA1 domain of hemagglutinin (HA). Among these nanobodies, Nb10 was found to exhibit high-affinity and broad-spectrum neutralization capacity against viruses of clades 2.3.2.1 and 2.3.4.4 under both *in vitro* and *in vivo* conditions. Surprisingly, Nb10 exhibited excellent efficacy against the recombinant viruses Re6/PR8, Re8/PR8, Re10/PR8, Re11/PR8, and Re14/PR8 of the subtype H5, with average half-maximal inhibitory concentrations ranging from 0.01 to 0.42 µg/mL in a microneutralization assay. Furthermore, the intratracheal administration of Nb10 resulted in remarkable prophylactic and therapeutic efficacy in mice. The findings herein reveal that the virus-neutralizing effect of Nb10 is achieved by obstructing viral entrance into host cells. Moreover, Western blot analysis and enzyme-linked immunosorbent assay revealed that Nb10 recognizes a conformational epitope located in the region spanning amino acid residues 50–271 of the protein HA1 displayed on the surface of yeast cells. The predicted structure of the binding pocket indicates that Nb10 recognizes the highly conserved receptor-binding site of HA1. Taken together, the current study offers valuable insights for the development of protective therapeutics with broad-spectrum activity and the design of broadly protective influenza vaccines.

**IMPORTANCE:**

HPAIVs of subtype H5 have raised substantial public health concerns regarding the potential for viral adaptation and sustained human-to-human transmission. The prevention and treatment of the disease are replete with numerous challenges due to frequent antigenic alterations in the virus. Nanobodies have significant potential for clinical applications and therapies owing to their small size and robust tissue-penetrating capabilities. Herein, we describe the identification of Nb10, a broad-spectrum virus-neutralizing and protective nanobody that is effective against the currently circulating H5 HPAIVs of clades 2.3.2.1 and 2.3.4.4. The intratracheal administration of Nb10 afforded significant protection in mice infected with the H5 virus. This result provides novel insights for the rational design of antiviral pharmaceuticals. Furthermore, an analysis of the binding site of the target protein HA1 may be useful for the development of more effective vaccinations against influenza viruses of the subtype H5.

## INTRODUCTION

Outbreaks of highly pathogenic avian influenza viruses (HPAIVs) of subtype H5 in poultry have exposed humans to potential public health threats besides causing significant economic losses (1). The avian influenza viruses (AIVs) of H5 have been categorized into nine distinct clades (clades 0–9) based on phylogenetic analyses of hemagglutinin (HA), and a few of these clades have been further subdivided into subclades (2). Clade 2.3.2.1 of H5 viruses was discovered in the wild birds of Hong Kong in 2007 (3). As per an assessment (4), the last reported fatal case of human infection with a virus of clade 2.3.2.1c was recorded in 2024. The H5 viruses of clade 2.3.4.4 have undergone rapid propagation via migratory wild aquatic birds since 2014 and have been subjected to genetic reassortment; these newly emerging HPAIVs are referred to as subtype H5NX (5, 6). Since 2020–2021, H5 viruses of clade 2.3.4.4b have caused extensive outbreaks in poultry and certain mammalian species. Furthermore, viruses of this clade have been detected in dairy cattle and unpasteurized milk specimens in several states of the USA. In March 2024, an adult employee of a dairy farm was found to be infected with an HPAIV of subtype H5 following exposure to infected cows (7). As per reports, 13 humans were found to be infected by viruses of clade 2.3.4.4b from the beginning of 2021 to April 2024 (4).

The primary methods currently employed for combating AIV infections are vaccinations and antiviral medications. Based on the strain circulating at the time, inactivated vaccines Re5, Re6, Re8, Re10, Re11, Re12, Re13, and Re14 have been produced and employed to reduce the impact of H5 HPAIV on the poultry industry in China since 2008. However, the manufacture of vaccines for newly emerging viruses is expected to involve a delay of at least several months, while the effectiveness of antiviral medications is limited owing to the development of resistance in viruses. Antibody therapy remains an important and feasible treatment option against viruses such as human immunodeficiency virus-1 (8), severe acute respiratory syndrome coronavirus (9), and Ebola virus (10). Numerous monoclonal antibodies (mAbs) that neutralize AIVs and target viruses of the ancestral H5Nx lineage have been reported to date (11, 12), but only a few antibodies are known to typically target viruses of circulating clades 2.3.4.4 or 2.3.2.1 (13–15).

Nanobodies have been used as diagnostic and therapeutic agents, given their desirable properties of high solubility, exceptional thermal stability, ease of production, and small size (16–24). Nanobody, otherwise known as the variable domain of heavy chain antibody (VHH), is the antigen-binding component of heavy chain-only antibodies that are generated by camelids and has a molecular mass of approximately 15 kDa (25). The nanobodies can penetrate the cavities of immunogens owing to their longer complementarity-determining region 3 (CDR3) and small size (26). This suggests that VHHs may be capable of targeting certain epitopes that are inaccessible to conventional antibodies; in turn, this provides valuable information for structure-based vaccine design. However, studies on nanobodies against circulating ancestral viruses of the subtype H5 or the presently circulating strains are limited in number (27). In addition, the majority of mAbs that have been investigated in animal studies are typically administered via intraperitoneal or intravenous routes. By contrast, an equivalent dosage of antibody or nanobody delivered via the intratracheal or intranasal routes has been shown to directly reach the respiratory epithelium, resulting in higher survival rates and significant body weight recovery compared to the mAbs administered via intraperitoneal or intravenous routes (18, 27–29). Therefore, the intratracheal or intranasal delivery of an antibody or a nanobody is likely to enhance its neutralization efficacy and reduce the required dose, resulting in lower costs and increasing the feasibility for clinical application.

HA is initially produced as a single polypeptide that is assembled into a trimeric structure known as HA0. During infection, host proteases cleave HA0 into its subunits, HA1 and HA2. The receptor-binding site (RBS) is a part of subunit HA1 and located in the globular head domain of the HA molecule; it is important for attachment with the host receptor and facilitates viral entry during influenza infection. Subunit HA2, along with several residues of subunit HA1, constitutes the conserved stem region of HA that enables the fusion of the viral and cellular membranes upon activation in the acidic environment of the endosomes (30).

The current study describes the screening and identification of the nanobody Nb10 using yeast two-hybrid (Y2H) assay; this is the first known nanobody to demonstrate neutralizing activity against viruses of clades 2.3.2.1 and 2.3.4.4 under both *in vitro* and *in vivo* conditions. The capacity of Nb10 to bind and neutralize the viruses as well as the underlying mechanisms of inhibition were investigated herein. The yeast surface display (YSD) system was employed for identifying the conformational epitopes of Re8-HA1 that are recognized by Nb10; the predicted model and subsequent analysis of the complex of Nb10 with the antigen revealed that Nb10 specifically binds the conserved residues within the RBS of Re8-HA1. Nb10 was found to exhibit excellent prophylactic and therapeutic protective efficacy, suggesting that it may be considered a prospective candidate drug against HPAIVs of the subtype H5.

## RESULTS

### Isolation of nanobodies against H5 AIVs using Y2H assay

**Fig 1:**
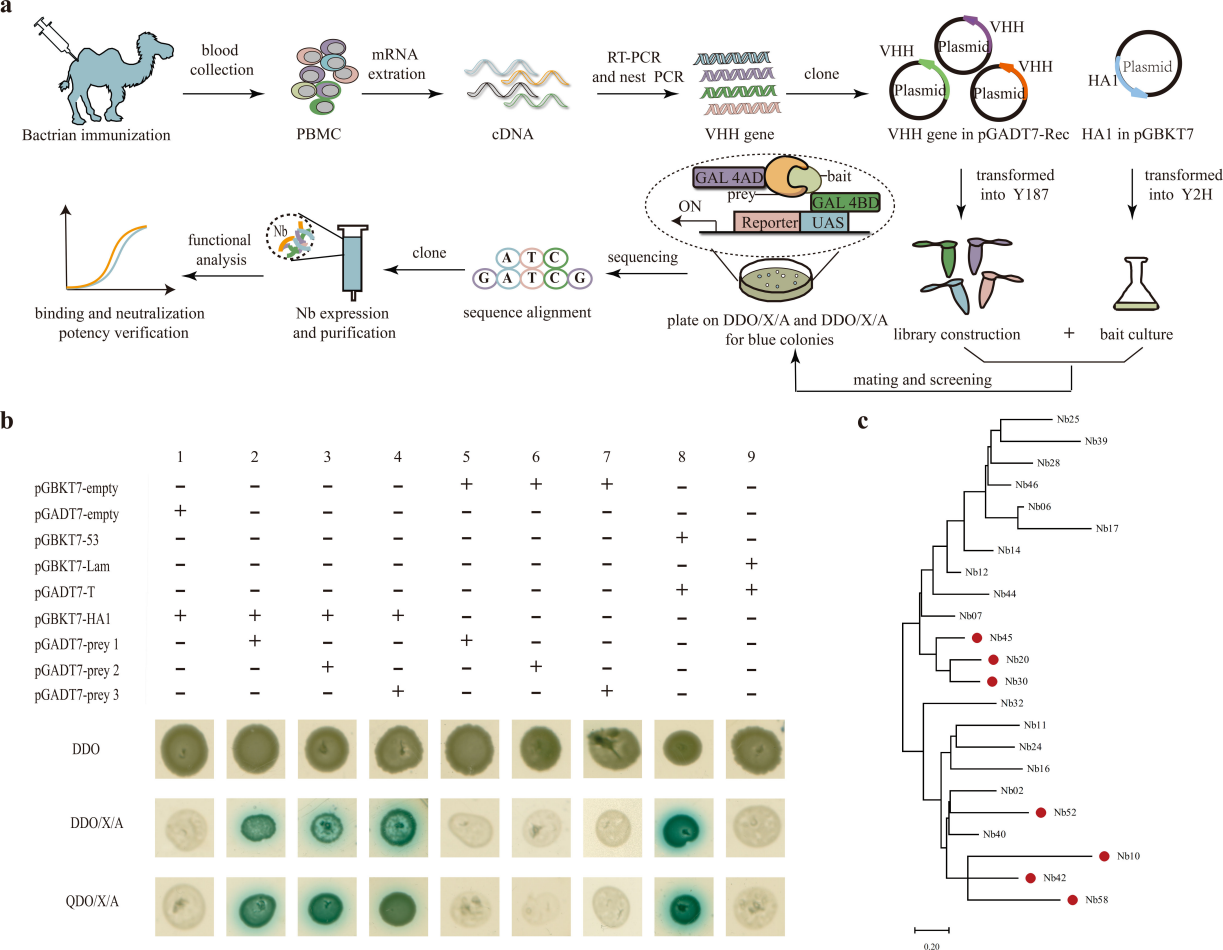
Nanobodies against H5-Re8 HA1 generation and selection process. (**a**) Schematic representation for the nanobody isolation against the influenza HA1 protein of H5-Re8. The positive clones were blued on DDO/X/A and QDO/X/A plates. DDO/X/A, SD medium lacking tryptophan and leucine but with X-α-gal and AbA; QDO/X/A, SD medium lacking adenine, histidine, tryptophan, and leucine but with X-α-gal and AbA. (**b**) Candidate nanobody screening by yeast two-hybrid mating assays. Positive protein–protein interactions appeared white in DDO plates and blue on DDO/X/A and QDO/X/A plates, while negative interactions resulted in no colonies on the DDO/X/A or QDO/X/A plates. The left panel used “+” to show the presence of the plasmid and “–” for its absence. (**c**) Phylogenetic tree based on the amino acid sequence. The phylogenetic tree was constructed from 23 VHH amino acid sequences by maximum likelihood in MEGA11. Nanobodies with significant binding capacity from the enzyme-linked immunosorbent assay are indicated by the red dot. The scale bar at the bottom corresponds to variation in the amino acid sequence. AbA, aureobasidin A; DDO/X/A, double dropout growth medium lacking tryptophan and leucine; PBMC, peripheral blood mononuclear cell; QDO/X/A, quadruple dropout growth medium lacking adenine, histidine, tryptophan, and leucine; RT-PCR, reverse transcription PCR; SD, synthetic dropout.

For the construction of the Y2H library, the VHH-encoding sequence was amplified from total RNA extracted from peripheral blood mononuclear cells (PBMCs) using two-step nested polymerase chain reaction (PCR) and inserted into the linearized vector pGADT7-Rec to generate recombinant plasmids. These plasmids were then introduced into Y187 yeast cells for the generation of the Y2H library (Fig. 1a). The library titer was assessed by counting the number of independent colonies and estimated at approximately (1.5 ± 0.06) × 10^9^ cfu/mL (Fig. S1a), reflecting a sufficient library size of (1.5 ± 0.06) × 10^11^ cfu (data not shown). An analysis of the transformants revealed that accurate insertion occurred in nearly 100% of the tested transformants (Fig. S1b). Furthermore, 10 colonies did not contain duplicated sequences (data not shown), demonstrating the successful construction of the prey library with a high diversity.

Prior to Y2H screening, the absence of autoactivation of the protein Re8-HA1 and its potential toxic effects in the yeast strain Y2HGold were evaluated (data not shown) (31). The transformants obtained using the bait plasmid and pGADT7-empty as a control for the screening (Fig. 1b, lane 1) indicated the absence of any interactions between the proteins encoded by pGBKT7-HA1 and pGADT7-empty. Screening of the zygotes (Fig. S1c) obtained upon mating the strains Y187 (harboring pGADT7-preys) and Y2HGold (harboring pGBKT7-HA1) for 20 h revealed a high mating efficiency. Following mating, the cells were subjected to double dropout growth medium lacking tryptophan and leucine (DDO/X/A) and subsequently, quadruple dropout growth medium lacking adenine, histidine, tryptophan, and leucine (QDO/X/A; please refer to Materials and Methods for further details), yielding a total of 50 blue-colored yeast colonies. These potential positive clones were subjected to sequencing analysis and found to correspond to 23 proteins (sequences are included in Table S1) that potentially interact with HA1. To distinguish genuinely positive interactions from the false-positive ones, the plasmids encoding these 23 proteins were individually cotransformed with pGBKT7-HA1 or pGBKT7-empty into the strain Y2HGold. As shown in lanes 2–4 of Fig. 1b, three randomly selected positive clones (cotransformants harboring pGADT7-preys and pGBKT7-HA1) appeared white on DDO plates but blue on both the DDO/X/A and QDO/X/A plates. All the cotransformants harboring pGADT7-preys and pGBKT7-empty appeared white on DDO plates but failed to yield any colonies on the DDO/X/A or QDO/X/A plates; three clones were randomly selected as representatives (Fig. 1b, lanes 5–7). These results further validate the genuine interactions between the VHHs and HA1. Lanes 8 and 9 of Fig. 1b correspond to the positive and negative control groups, respectively.

Subsequently, the recombinant plasmid, pPICZαA, containing the genes encoding the nanobodies fused with the 6x-His-tag or Twin-Strep-tag was employed for the transformation of *Pichia pastoris* strain X33, and the expressed nanobodies were purified. The binding capacity of the purified nanobodies was further confirmed via indirect enzyme-linked immunosorbent assay (ELISA) using immobilized and inactivated H5-Re8 virus; seven of the selected nanobodies yielded noticeably higher values of optical density (OD) than the remaining 16 nanobodies, as depicted in Fig. S1d. Sodium dodecyl sulfate-polyacrylamide gel electrophoresis (SDS-PAGE) analysis revealed that these seven nanobodies had molecular weights of approximately 15 kDa (Fig. S1e) and purity of approximately 95%. The maximum likelihood method was employed for phylogenetic analysis based on the amino acid sequences of the 23 isolated nanobodies. The isolated nanobodies with significant binding capacity for inactivating viruses have been highlighted with a red dot (Fig. 1c).

### Characterization of the nanobodies against H5 HA1

**Fig 2:**
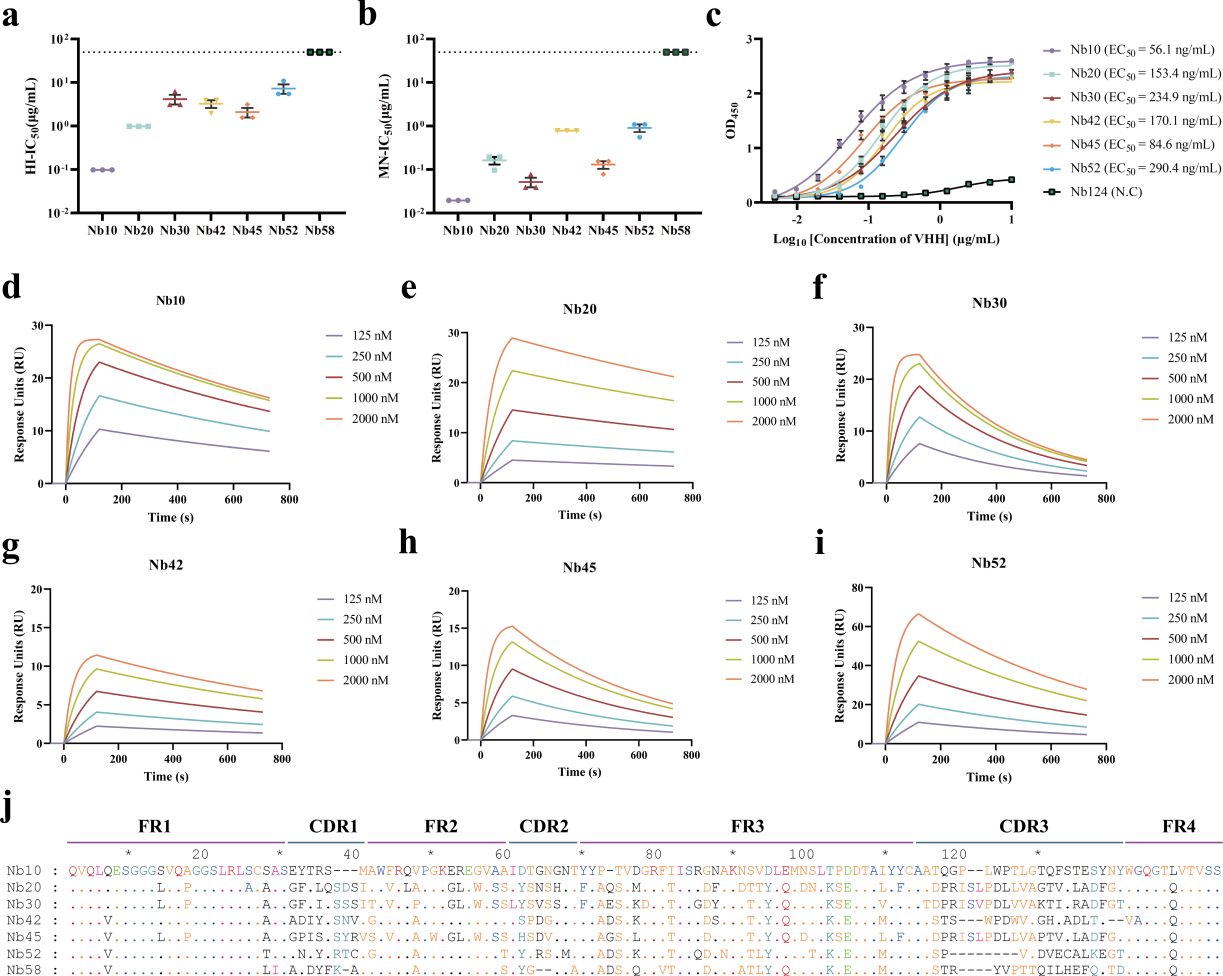
Characterization of anti-H5 HA1 nanobodies. (**a**) Hemagglutination inhibition (HI) titers of nanobodies against the Re8/PR8 virus. The dashed line indicates 50 µg/mL. Data were expressed as mean ± SEM (*n* = 3). (**b**) *In vitro* microneutralization of nanobodies against the Re8/PR8 virus. Half-maximal inhibitory concentration (IC_50_) was detected by an immunofluorescence assay. The dashed line indicates 50 µg/mL. The IC_50_ values were expressed as mean ± SEM (*n* = 3). (**c**) Half-maximal effective concentrations (EC_50_ values) measured by ELISA. Nb124 did not bind to the antigen. EC_50_ values were calculated by four-parameter non-linear regression. Data were plotted as mean ± SEM (*n* = 3). (d–i) Surface plasmon resonance measurements of each nanobody’s binding kinetics to Re8-HA1. The interaction responses collected for HA1 and nanobodies were noted as response units (RU). The data points of binding affinity were processed by Prism GraphPad. (**j**) Sequence alignment of neutralizing nanobodies. The deduced amino acid sequences were given in single-letter codes. Dashed lines denote the absence of amino acid residues; dots indicate residues identical to clones in Nb10. The amino acid residues were numbered, and the complementarity-determining regions (CDRs) and framework regions (FRs) were labeled.

The nanobodies so identified were further characterized using hemagglutination inhibition (HI) and microneutralization (MN) assays. Nanobody Nb10 exhibited strong activity against strain Re8/PR8 in both HI and MN assays, with average half-maximal inhibitory concentrations (IC_50_) of 0.10 ± 0.00 and 0.02 ± 0.00 µg/mL, respectively (Fig. 2a and b; Table S2). The other nanobodies, Nb20, Nb30, Nb42, Nb45, and Nb52, exhibited HI activities with average IC_50_ values of 0.98 ± 0.00, 4.17 ± 1.04, 3.26 ± 0.65, 2.08 ± 0.52, and 7.29 ± 1.82 µg/mL, respectively (Fig. 2a; Table S2). These nanobodies also exhibited potent neutralizing activity against Re8/PR8, with MN-IC_50_ values of 0.16 ± 0.03, 0.05 ± 0.01, 0.78 ± 0.00, 0.13 ± 0.03, and 0.91 ± 0.18 µg/mL, respectively (Fig. 2b; Table S2). However, nanobody Nb58 failed to exhibit detectable activities against strain Re8/PR8 in the HI and MN assays even at the highest concentration tested (50 µg/mL; Fig. 2a and b; Table S2). The binding affinities of these neutralizing nanobodies against Re8-HA1 were further evaluated using indirect ELISA and surface plasmon resonance (SPR). The half-maximal effective concentrations (EC_50_) of Nb10, Nb20, Nb30, Nb42, Nb45, and Nb52 against Re8-HA1 ranged from 56.1 to 290.4 ng/mL (Fig. 2c; Fig. S2a). As expected, Nb10 had the lowest EC_50_ value of 56.1 ng/mL, indicating stronger binding than that of the other nanobodies. Additionally, an analysis of the kinetics of binding of the nanobodies with Re8-HA1 revealed equilibrium dissociation rate constants (*K*_*D*_) ranging from 1.21E−07 to 9.93E−08, as shown in Fig. 2d through i and Table 1. By contrast, the control nanobody Nb124 failed to bind Re8-HA1 (Fig. S2b). The sequences of the seven nanobodies were aligned and marked, as shown in Fig. 2j. The framework regions were conserved among these sequences, while the amino acids in the CDRs were considerably distinct.

**TABLE 1:** Binding kinetics of the nanobodies to Re8-HA1^*a*^

Antigen	Analyte	*k*_*a*_ (1/Ms)	*k*_*d*_ (1/s)	*K*_*D*_ (M)
Re8-HA1	Nb10	3.30E+04	8.56E−04	2.60E−08
Re8-HA1	Nb20	1.05E+04	5.14E−04	4.65E−08
Re8-HA1	Nb30	1.84E+04	2.81E−03	1.53E−07
Re8-HA1	Nb42	3.03E+04	8.64E−04	2.85E−08
Re8-HA1	Nb45	1.90E+04	1.89E−03	9.93E−08
Re8-HA1	Nb52	1.19E+04	1.44E−03	1.21E−07

^*a*^ Binding kinetics of nanobody candidates to Re8-HA1 proteins. *k*_*a*_, association rate constant; *k*_*d*_, dissociation rate constant; *K*_*D*_, equilibrium dissociation rate constant. *K*_*D*_ = *k*_d_/*k*_a_.

### Broad-spectrum neutralization and binding profiles of Nb10

**Fig 3:**
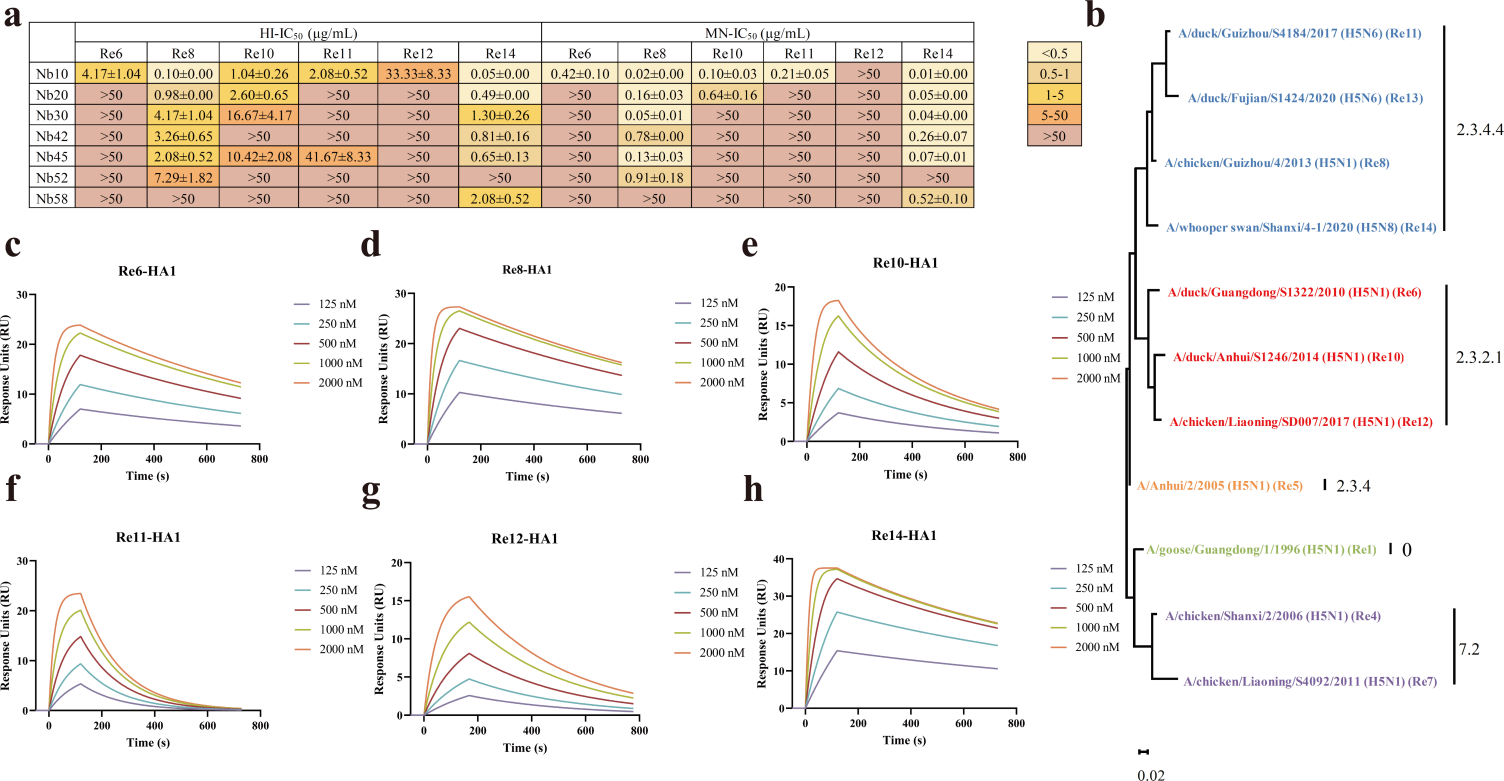
Neutralization and binding profile of Nb10. (**a**) The summary of IC_50_ of the nanobodies. The range of average IC_50_ values determined by the HI and MN assays is highlighted in color based on the mean according to the color bar of the right panels. The experiment was carried out in triplicate, and data were presented as mean ± SEM (*n* = 3). (**b**) Phylogenetic tree of amino acid sequences of HA from commercially inactivated vaccine viruses. The evolutionary tree was built with the maximum likelihood method in MEGA11. Candidate vaccine viruses and their associated H5 clade numbering are shown next to the branches. The scale bar represents a 2% difference in amino acid sequence. (c–h) Binding kinetics of Nb10 with different HA1s measured by SPR. The concentrations of the Nb10 are indicated in the inset of the figures. The figures were generated by GraphPad Prism.

The broad-spectrum neutralization activities of these selected nanobodies were verified by employing a diverse panel of recombinant H5 viruses (Table S3), including those of clades 2.3.2.1 (strains Re6/PR8, Re10/PR8, and Re12/PR8) and 2.3.4.4 (strains Re8/PR8, Re11/PR8, and Re14/PR8). Surprisingly, most of the nanobodies showed stronger activities against Re14/PR8 than Re8/PR8 in both HI and MN assays, with Nb10 exhibiting IC_50_ values of 0.05 ± 0.00 and 0.01 ± 0.00 µg/mL, respectively, against Re14/PR8. Moreover, Nb58 also exhibited the capacity to neutralize Re14/PR8 in both HI and MN assays, with average IC_50_ values of 2.08 ± 0.52 and 0.52 ± 0.10 µg/mL (Fig. 3a), respectively. However, Nb52 failed to neutralize virus Re14/PR8 in either assay (Fig. 3a). The remaining nanobodies neutralized viruses Re8/PR8 and Re14/PR8 in both assays at low concentrations (Fig. 3a). Most of the nanobodies were ineffective in neutralizing viruses Re6/PR8, Re10/PR8, or Re11/PR8 in the MN assay, except for Nb10, which exhibited average IC_50_ values of 0.42 ± 0.10, 0.10 ± 0.03, and 0.21 ± 0.05 µg/mL, respectively. The average IC_50_ values of Nb10 against viruses Re6/PR8, Re10/PR8, Re11/PR8, and Re12/PR8 in the HI assay were found to be 4.17 ± 1.04, 1.04 ± 0.26, 2.08 ± 0.52, and 33.33 ± 8.33 µg/mL (Fig. 3a). Taken together, these results suggest that Nb10 exhibits activity against viruses of both clades 2.3.2.1 and 2.3.4.4 (Fig. 3b). Therefore, subsequent experiments were focused on further characterization of Nb10. The binding properties of Nb10 with a panel of recombinant HA1 derived from viruses of clades 2.3.2.1 (including Re6-HA1, Re10-HA1, and Re12-HA1) and 2.3.4.4 (including Re8-HA1, Re11-HA1, and Re14-HA1) were subsequently assessed (Fig. S3a and b; Table S4). Nb10 was found to exhibit high-affinity binding with all the tested HA1, with *K*_*D*_ values of 4.50E−08, 1.24E−07, 3.05E−07, 3.07E−07, and 1.59E−08 for Re6-HA1, Re10-HA1, Re11-HA1, Re12-HA1, and Re14-HA1, respectively (Fig. 3c through h; Table 2). All primers, plasmids, and strains used above are listed in Tables S5 to S7 (please refer to Materials and Methods for further details).

**TABLE 2:** Binding kinetics of the Nb10 to HA1s^*a*^

Antigen	Analyte	*k*_*a*_ (1/Ms)	*k*_*d*_ (1/s)	*K*_*D*_ (M)
Re6-HA1	Nb10	2.42E+04	1.09E−03	4.50E−08
Re8-HA1	Nb10	3.30E+04	8.56E−04	2.60E−08
Re10-HA1	Nb10	2.78E+04	3.44E−03	1.24E−07
Re11-HA1	Nb10	2.20E+04	6.72E−03	3.05E−07
Re12-HA1	Nb10	9.07E+03	2.77E−03	3.07E−07
Re14-HA1	Nb10	6.18E+04	9.85E−04	1.59E−08

^*a*^ SPR measurements of Nb10 to different clades of H5-HA1 proteins, showing the equilibrium dissociation constant (*K*_*D*_), the association rate constant (*k*_*a*_), and the dissociation rate constant (*k*_*d*_), with *K*_*D*_ calculated as *k*_*d*_/*k*_*a*_.

### Prophylactic and therapeutic efficacy of Nb10 against H5 viruses under *in vivo* conditions

**Fig 4:**
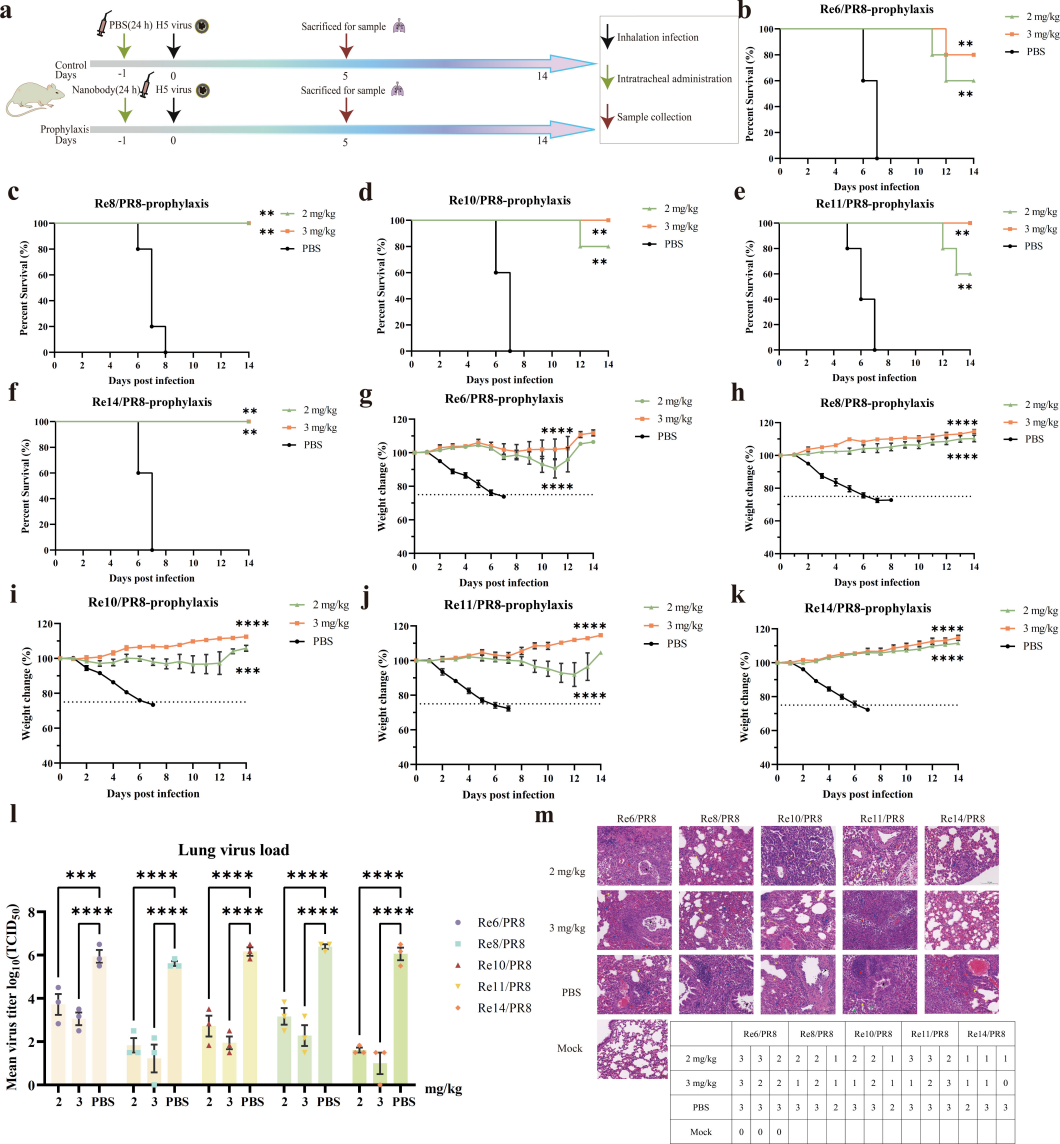
Prophylactic efficacy of Nb10 *in vivo*. (**a**) The experimental design for prophylactic efficacy study. The black, green, and red arrows indicate the intranasal infection, the intratracheal administration, and the lung collection, respectively. (b–k) The percentage of initial body weight and survival curves. The survival rates and weight-loss statuses of the mice (*n* = 5) were measured daily for 14 days after inoculation (day 0). PBS was used for the control group. Statistical analysis for comparison of weight change was processed in one-way analysis of variance (ANOVA), and survival analysis used the Kaplan-Meier method performed in GraphPad Prism. Data points in the graph represent the mean ± SEM (*n* = 5). (**l**) Lung virus titers at 5 dpi with indicated treatments. The virus load of each mouse (*n* = 3) was determined by tissue culture infectious dose at 50% (TCID_50_) assays. Data points in the graph represent the mean ± SEM (*n* = 3). Statistical analysis for comparison of virus load was performed using a two-way ANOVA. Statistical significance is represented as **P < 0.01, ****P < 0.001, ****P < 0.0001. (**m**) Representative images of hematoxylin and eosin and evaluation of histopathological lesion. Lymphocytes and macrophages were indicated in blue arrows, inflammatory cells in red arrows, widening of the alveolar septa in yellow arrows, erythrocytes and serous exudate in green, and exfoliated epithelial cells in black. The grades of lung sections, corresponding to each individual mouse, were classified into 0–3 based on area percentages of histopathological abnormalities: 0, no lesions; 1, minimal (1%–20%); 2, moderate (21%–20%); 3, severe (51%–100%).

For the evaluation of the prophylactic efficacy of Nb10, the mice received a single dose of Nb10 or phosphate-buffered saline (PBS) intratracheally 24 h prior to the intranasal challenge with the virus; lungs were collected on the fifth day post−viral infection (dpi) for further analysis (Fig. 4a). A survival rate of 80% was observed at a dose of 3 mg/kg for the Re6/PR8 virus (Fig. 4b), while 100% protection against all the tested H5 viruses was provided by Nb10 (Fig. 4c through f). Moreover, all the groups treated with Nb10 showed an increase in body weight over a duration of 2 weeks (Fig. 4g through k). Remarkably, a dose as low as 2 mg/kg still afforded 100% protection against both Re8/PR8 and Re14/PR8 (Fig. 4c and f) and 80% protection against Re10/PR8 (Fig. 4d), with significant differences (*P* < 0.0001, *P* < 0.0001, and *P* < 0.001, respectively) in weight fluctuations compared with that of the corresponding control groups (Fig. 4h, i, and k). Furthermore, the viral titers in the lungs of treated mice were significantly reduced compared to those in the control mice at 5 dpi (Fig. 4l), reflecting the prophylactic efficacy of Nb10. Additionally, the viral load detected in the lung homogenates was found to be inversely proportional to the concentration of Nb10 employed for the treatment of mice, with higher concentrations of Nb10 resulting in lower viral loads. The extent of lung lesions in mice was scored based on the percentage area of histopathological abnormalities. As shown in Fig. 4m, Nb10 alleviated lung injury, characterized as fewer areas showing widened alveolar septa and congestion, a significant decrease in inflammatory cells, lower infiltration of lymphocytes around the blood vessels, fewer macrophages in the alveolar spaces, and reduced exfoliation of cells and exudates in the bronchi compared to those in the PBS control group. These observations are consistent with the decreased viral load and reduced weight loss observed in the treated mice, reflecting the potency of the protection afforded by Nb10 against all the tested H5 viruses. Significant abnormalities were not detectable in mock-infected mice.

**Fig 5:**
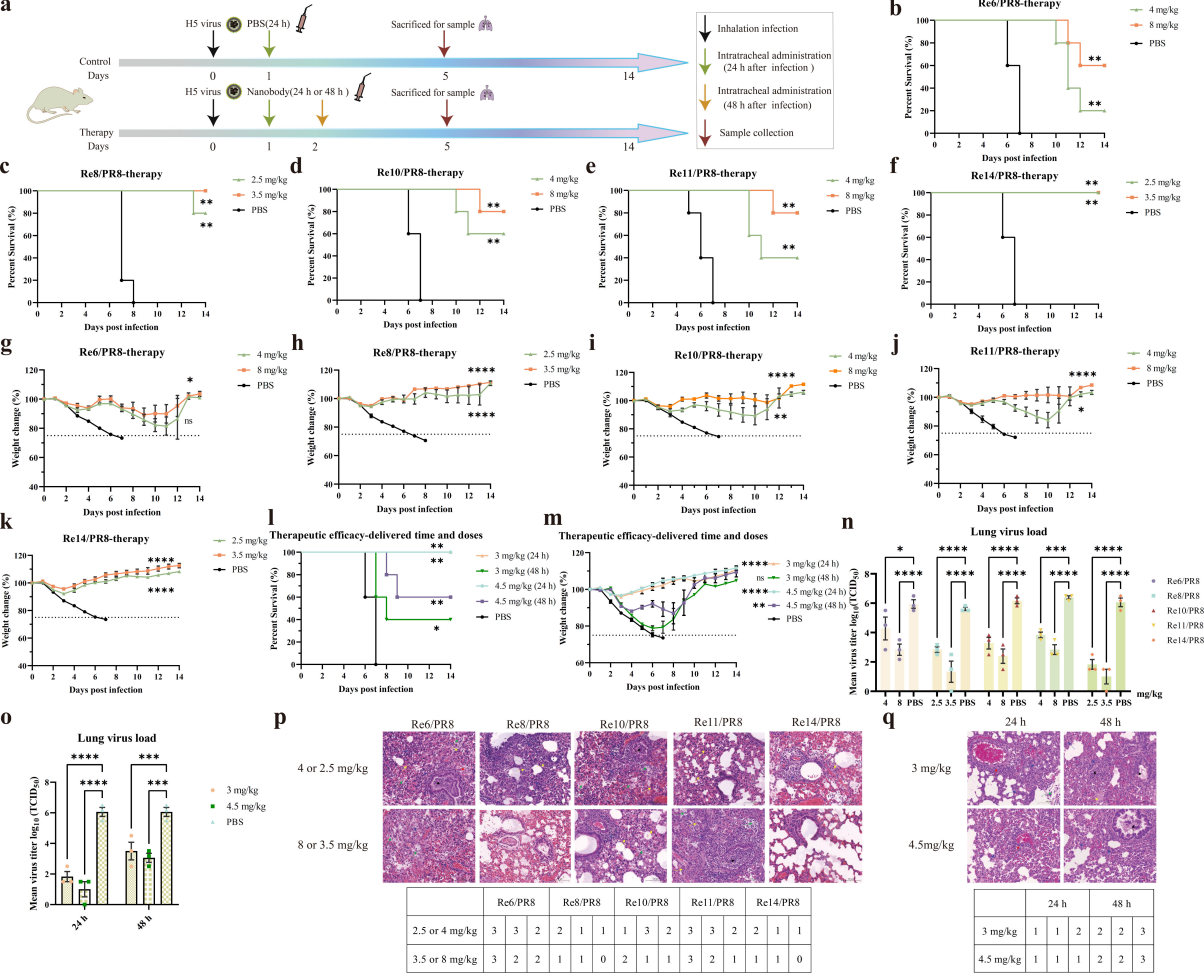
Therapeutic efficacy of Nb10 *in vivo*. (**a**) The experimental design for therapeutic efficacy study. The black, green, yellow, and red arrows indicate the intranasal infection, the intratracheal administration, and the lung collection, respectively. The green arrow signifies dosing at 24 h post-infection, whereas the yellow arrow denotes dosing at 48 h post-infection. (b–m) The weight-loss and survival curves of BALB/c mice. Mice of (*n* = 5 per group) panels b–k were administered 24 h after being challenged. Mice of panels l and m were dosed at 24 or 48 h post-infection with a single dose of 3.0 or 4.5 mg/kg. The survival rates and weight-loss statuses of the mice were monitored until 14 days after infection. The administration of PBS at 24 h after infection served as the negative control. Data are presented as the means ± SEM. Weight change analysis was processed in one-way ANOVA, and survival analysis was performed by the Kaplan–Meier method. (**n and o**) The viral load of the half lungs. Lung virus titers of panel n from mice treated 24 h post-infection and panel o from mice treated identically as in panels l and m. The lung viral titers of three mice were measured by the TCID_50_ assay. Statistical significance was determined by using two-way ANOVA with GraphPad. Statistical significance is indicated as follows: *P < 0.05, **P < 0.01, ***P < 0.001, ****P < 0.0001; ns, no significance. (**p and q**) Representative images of H&E and lung histology analysis. The samples of panel *P* from mice treated 24 h post-infection and panel q from mice treated identically as in panels l and m. Inflammatory cells, widening of the alveolar septa, erythrocytes, and serous exudate, and exfoliated epithelial cells are indicated in blue, yellow, green, and black arrows, respectively. Based on area percentages of abnormalities, the histopathological grades were classified into 0–3: 0, no lesions; 1, minimal (1%–20%); 2, moderate (21%–20%); and 3, severe (51%–100%).

For the evaluation of therapeutic efficacy, different doses of Nb10 were administered 24 or 48 h following the infection of mice, and the lungs were collected at 5 dpi (Fig. 5a). The administration of 4 or 8 mg/kg of Nb10 was found to afford partial protection to mice challenged with viruses Re6/PR8 (20% and 60%, respectively; Fig. 5b), Re10/PR8 (60% and 80%, respectively; Fig. 5d), and Re11/PR8 (40% and 80%, respectively; Fig. 5e). Additionally, the mice treated with Nb10 exhibited a slight increase in body weight over a period of 2 weeks (Fig. 5g, i and j). Furthermore, the protective effects appeared to be enhanced with an increase in the dosage of Nb10. Astonishingly, the administration of Nb10 at doses of 2.5 and 3.5 mg/kg afforded a high degree of protection and decreased the morbidity as well as mortality associated with viruses Re8/PR8 (80% and 100%, respectively; Fig. 5c and h) and Re14/PR8 (100% and 100%, respectively; Fig. 5f and k). Therefore, the therapeutic efficacy of Nb10 against Re14/PR8 was further evaluated by administering a fixed dose of Nb10 at different time points. The administration of either 3.0 or 4.5 mg/kg of Nb10 24 h after the infection of mice with virus Re14/PR8 resulted in complete protection and only a minor weight loss, which was followed by progressive recovery from day 4 (Fig. 5l and m). Conversely, the administration of Nb10 (3.0 or 4.5 mg/kg) 48 h postviral challenge provided only moderate protection, resulting in survival rates of 40% and 60% for the respective doses (Fig. 5l and m). Moreover, protective efficacy was highly effective when administered during the early stages of infection of Nb10 but not afforded when the treatment was delayed to day 3 post-infection (data not shown). In all the abovementioned experiments on therapeutic efficacy, the viral titers and histopathological lesions in the lung were reduced to varying degrees and in certain instances, even to below the limits of detection compared to those in the corresponding control groups (Fig. 5n through q). These results are consistent with the data on survival and body weight fluctuations of mice subjected to the corresponding treatments. Overall, these results indicate that Nb10 can afford mice with broad protection against viruses of clades 2.3.2.1 and 2.3.4.4 under *in vivo* conditions.

### The antiviral efficacy of Nb10 is mediated via the inhibition of viral attachment to host cells

**Fig 6:**
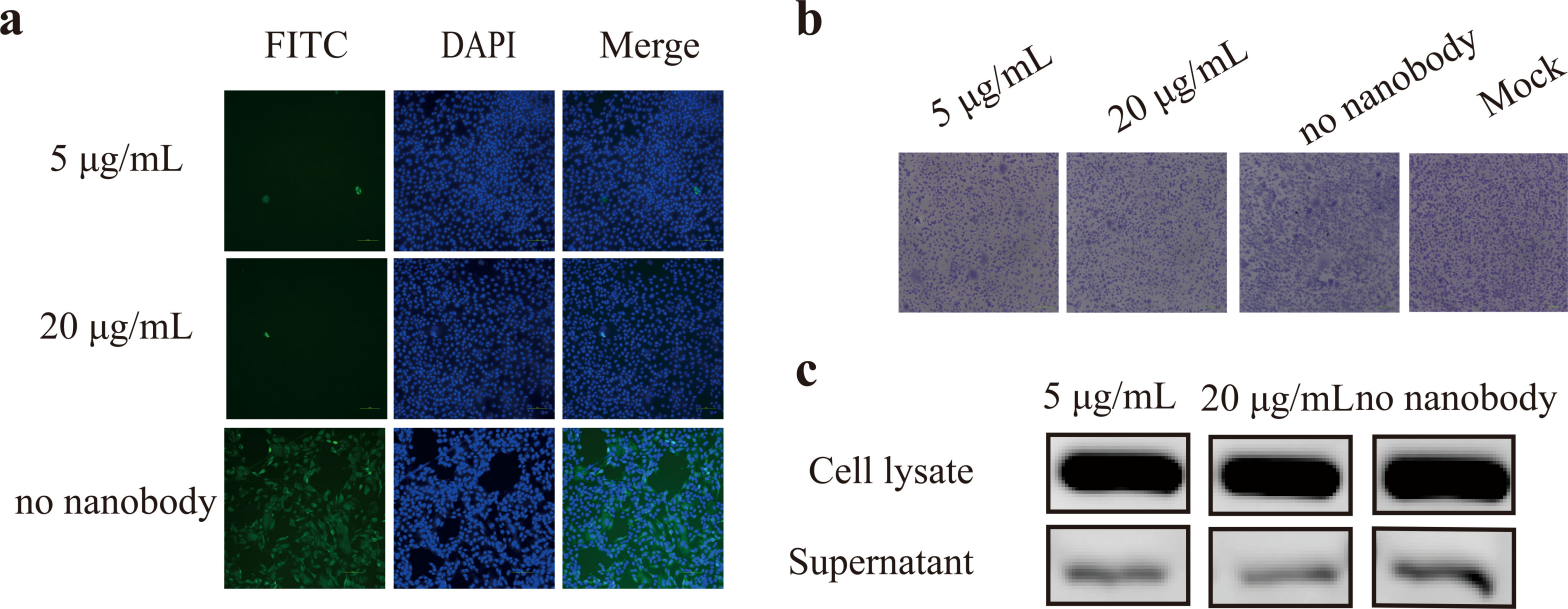
Neutralization mechanisms of Nb10. (**a**) Cell-based attachment inhibited by Nb10. The nucleoprotein (NP) of influenza A was visualized by fluorescein isothiocyanate (FITC) (green)-conjugated secondary antibodies. No influenza NP signal was observed in both 5 and 20 µg/mL and an intense signal of NP in cells infected by a non-mixed virus. No antibody pre-incubated with the virus was used as a negative control. FITC, green, infected cells positive for NP protein; 4′,6-diamidino-2-phenylindole (DAPI), blue, staining for nucleus; merge, two fluorescent signals distinguished at the same location, overlapped, or not in a cell. Results from one representative of two independent experiments are shown. (**b**) Nb10 did not interfere with the formation of syncytia. The cells were stained with Giemsa. Syncytia were present in similar numbers on treated or untreated (negative control) cells in three fields counted by three people, and mock showed no syncytium in the non-infected by the H5 virus. Therefore, Nb10 was unable to prevent the low pH-induced conformational change and the further formation of syncytia. Representative images of three independent experiments are shown. (**c**) Nb10 not involved in the release of progeny virions. The viral progeny from infected cells was quantified by the expression of NP. No nanobody treated served as a negative control. The virus was detected in all supernatants and lysates of the Madin–Darby canine kidney cells with a similar intense band compared to the control, indicating that Nb10 showed no impact on the release of progeny virions. Results from one representative of two independent experiments are shown.

The replication of the virus following internalization into cells was quantitated by monitoring the levels of influenza nucleoprotein (NP) using immunofluorescence assay (IFA). As shown in Fig. 6a, the immunofluorescence corresponding to NP was undetectable when the virus was pretreated with Nb10 at either of the tested doses (5 and 20 µg/mL), whereas an intense signal was observed in the negative control where the nanobody was not pre-mixed with the virus. Taken together, these results suggest that Nb10 affords protection against viral infection by preventing the entry of the virus into host cells. The assay for the inhibition of cell–cell fusion failed to reveal statistically significant differences in the average numbers of syncytia per field between the groups with and without Nb10 treatment (Fig. 6b). These results confirm that Nb10 does not affect HA-mediated membrane fusion and the following formation of syncytia. Furthermore, Western blot (WB) analysis revealed that similar concentrations of NP were detectable in the cell lysate supernatants of all groups, including those incubated with different concentrations of Nb10 and the untreated control. Nb10 was found to be incapable of disrupting the release of the virus (Fig. 6c).

### Identification of the epitope recognized by Nb10 and structural analysis of the Re8-HA1:Nb10 complex

**Fig 7:**
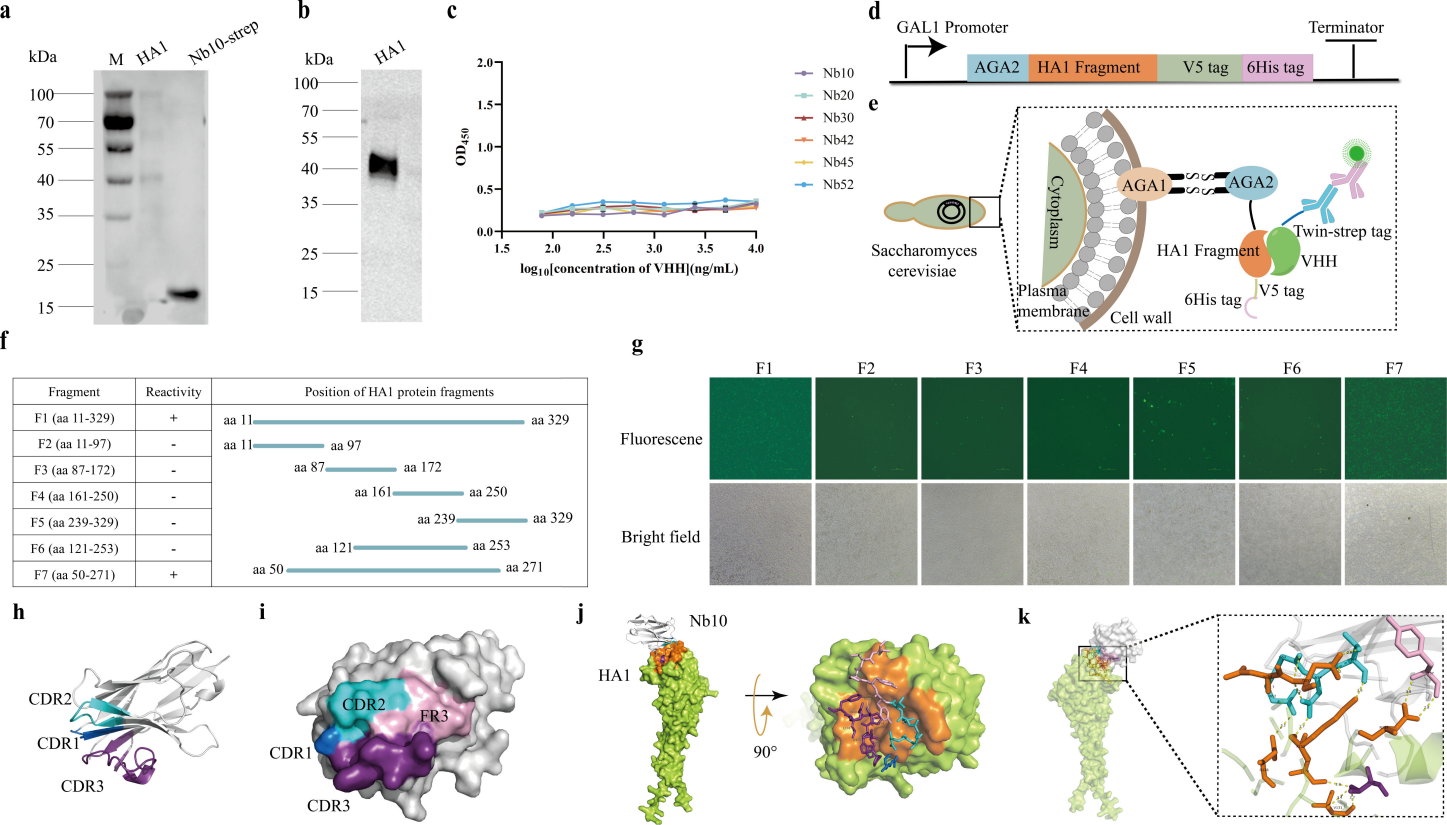
Epitope mapping and structural prediction. (**a**) Conformation-dependent epitope identification. M denotes the 180 kDa plus pre-stained protein marker. HA1 protein and Nb10 with twin-strep tag were loaded. Nb10 could not react with the denatured HA1 protein, and no signal was shown. The Nb10 with strep tag served as control. (**b**) The denatured HA1 proteins. A band at approximately 40 kDa is shown. (**c**) All nanobodies targeting the overlapping epitopes or spatially adjacent epitopes. No nanobodies were bound to the antigen when Nb10 pre-captured the HA1, according to the OD value. Data were expressed as mean ± SEM (*n* = 3). (**d**) Vector construction for the interested protein displayed by *Saccharomyces cerevisiae* EBY100. AGA2, HA1 fragments, V5 tag, and 6x-His-tag are shown in blue, orange, green, and purple, respectively. (**e**) Schematic representation of yeast surface display. The fusion protein was subsequently secreted to the extracellular space and can be detected. (**f**) Schematic drawing of Nb10 against HA1 and its fragments corresponding to the region spanning amino acid residues of HA1. The HA1 fragments were diagrammed. “+” and “–” indicate the reacting and non-reacting in the IFA assays. (**g**) Reactivity of Nb10 against HA1-truncated protein by IFA. Labels on top of each image indicate the different fragments of HA1. (**h**) Three-dimensional structure prediction of Nb10. CDRs were labeled and marked with different colors. The cartoon represented CDR1 in marine, CDR2 in cyan, and CDR3 in violet purple. (**i**) Surface representation of the Nb10 binding site. Binding residues in CDR1, CDR2, FR3, and CDR3 are in marine, cyan, light pink, and violet purple, respectively. (**j**) Surface representation of the HA1 binding site. All epitopes on HA1 involved in binding with Nb10 are shown in orange. (**k**) The key residues involved in hydrogen bonds. The surface represented HA1 in lemon and Nb10 in gray. The interaction residues from HA1 (orange) and Nb10 (marine, cyan, light pink, and violet purple) were represented by sticks, and the key residues were labeled on the panel. The hydrogen bonds were represented by yellow dashed lines.

The specific types of epitopes recognized by Nb10 were identified by WB analysis using the baculovirus-expressed recombinant protein Re8-HA1. As shown in Fig. 7a, the reduced form of the HA1 protein was not recognized by Nb10, suggesting that non-linear epitopes are involved in the binding. Based on the demonstration of the highest binding affinity for HA1 in ELISA, the candidate antigenic epitope recognized by Nb10 was defined as a conformation-dependent epitope. The Strep-tagged Nb10 (Fig. 7a) and recombinant protein HA1 (Fig. 7b) were employed as controls. ELISA was employed for further characterizing the epitopes recognized by these six neutralizing nanobodies against Re8/PR8. All the OD values were low and did not exhibit any apparent increase upon increasing the concentration of the added nanobodies (Fig. 7c), suggesting that the attachment of Nb10 with HA1 precluded subsequent binding of the nanobodies with the antigen. These results suggest that all the nanobodies recognize overlapping epitopes or regions that are located in close proximity to one another, which is in line with the predicted structure obtained using the online resource AlphaFold 3 (Fig. S4).

To further define the epitopes recognized by Nb10, eukaryotic expression plasmids containing full-length and numerous truncated HA1 sequences with V5 and His-tag at the C-termini were generated. The GAL1 promoter regulating protein expression in the YSD system is suppressed in the presence of glucose but stimulated by galactose (Fig. 7d). Herein, the protein HA1 and its truncated fragments (the number of residues based on the numbering of HA from H3 viruses) were genetically linked with *Aga2* and presented on the surface of *Saccharomyces cerevisiae* EBY100 cells (Fig. 7e and f). As expected, Nb10 recognized the full-length (F1, aa 11–329) HA1 (Fig. 7g, lane 1), reflecting the successful display of the protein on the cell surface. By contrast, fluorescence signals were not detectable for the truncated protein fragments F2 (aa 11–97), F3 (aa 87–172), F4 (aa 161–250), and F5 (aa 239–329; Fig. 7g, lanes 2–5). These results revealed that none of the truncated fragments were recognizable by Nb10. Thus, truncated HA1 fragments of a larger size were employed for epitope screening, including the fragments F6 (aa 121–253) and F7 (aa 50–271). The fluorescence signal was undetectable in fragment F6 but evident in fragment F7 (Fig. 7g). These results demonstrate that the region comprising aa 50–271 on the surface of HA1 is recognized by Nb10, which is consistent with the predicted structure obtained using AlphaFold 3 (Table S8).

To locate the region on HA1 to which Nb10 binds, the three-dimensional (3D) structures of the HA1:Nb10 complex were predicted using AlphaFold 3. The model’s confidence was assessed using the predicted local distance difference test (pLDDT) on a scale of 0–100, with higher folding scores signifying higher confidence and varied along a protein chain. A very high (>90) pLDDT score was obtained for HA1 and Nb10 (data not shown). The predicted template modeling and interface predicted template modeling scores, used to measure the accuracy of the structure, were found to be 0.82 and 0.9 for the HA1:Nb10 complex, respectively (Table S9). The CDR of the modeled Nb10 is depicted in Fig. 7h, with CDR1, CDR2, and CDR3 presented in marine, cyan, and violet purple, respectively. Furthermore, the epitope on the interface has been highlighted in different colors in Fig. 7i and j. The predicted structure revealed that the interface of Nb10 with HA1 is mainly composed of CDR2 (D49, N52, G53, N54, and T55; cyan) and CDR3 (P98, L99, W100, P101, T102, L103, Q106, and F107; violet purple), as also one and five amino acids of CDR1 (R29, marine) and FR3 (Y56, Y57, P58, T59, and V60; light pink), respectively (Fig. 7i and j; Table S8). The abovementioned residues were found to interact with the highlighted orange residues of HA1 (numbering is based on the sequence of HA from H3 viruses): Y98, D131, S133, L133a, G134, V135, S136, A137, P145, W153, I155, K156, K157, N158, D159, A188, A189, E190, T192, N193, L194, and Q226 (Fig. 7j; Table S8). Among these, residues Y98, S133, G134, V135, S136, W153, K156, K157, E190, L194, and Q226 are highly conserved (>95%) among all H5 isolates (*n* = 3,000; data collected prior to November 2024, from the Influenza Database of the National Center for Biotechnology Information and the EpiFlu Database of the Global Initiative on Sharing All Influenza Data), as listed in Table S10. A total of 14 hydrogen bonds formed between Nb10 and HA1 are depicted by a yellow dashed line in Fig. 7k and involve key residues D131, L133a, V135, K156, N158, D159, and N193 of HA1 as well as N52, G53, N54, T55, Y57, and T102 of Nb10 (Table S11). Based on the relative positions on the amino acid sequence of HA, the RBS of HA is composed of the 130-loop, 150-loop, 190-helix, and 220-loop. In the current study, the binding sites for Nb10 in the globular head of HA1 were identified as the 130-loop, 150-loop, and 190-helix. The epitopes on Re8-HA1 or Re14-HA1 that are recognized by the other nanobodies obtained herein are depicted in Fig. S4, and details of the interaction are listed in Tables S9 and S10.

## DISCUSSION

The HPAIVs of subtype H5, and specifically, the viruses of the recently emerged and currently circulating clades 2.3.2.1 and 2.3.4.4, pose a significant risk to public health and increase the possibility of human infections (3, 32, 33). The emergence of new H5 variants may reduce the effectiveness of the available influenza vaccines and facilitate viral escape from the neutralizing antibodies against the ancestral strains (2, 3, 5, 34). Nanobodies have been developed for the prevention and treatment of severe acute respiratory syndrome, especially since the coronavirus disease 2019 pandemic (18). However, research on antibodies as well as nanobodies against viruses of circulating clades 2.3.2.1 and 2.3.4.4 remains limited (11, 15, 35). The present study describes the identification of a nanobody and the evaluation of its binding and neutralization capacities against the HPAIVs of subtype H5. Nb10 not only exhibited strong activities against the H5 viruses of clades 2.3.2.1 and 2.3.4.4 in the HI and MN assays but also afforded mice with robust protection when administered via the intratracheal route. These features of Nb10 support the premise that this nanobody is a highly promising candidate for therapeutic applications and that the Nb10-binding sites on HA1 may provide an exceptional template for the development of vaccines.

Herein, Nb10 was found to afford protection to the host cells by obstructing viral attachment, indicating that it may recognize a conformation-dependent epitope located in the RBS region. Indeed, the predicted structure obtained using AlphaFold 3 indicated that Nb10 may interact with the 130-loop, 150-loop, and 190-helix of the RBS region of HA. More importantly, the conservation of the nanobody-binding site on HA1 may explain the broad-spectrum activity of Nb10 against viruses of clades 2.3.2.1 and 2.3.4.4. The predicted structure of the HA1:Nb10 complex revealed that an eight-residue segment in the CDR3 of Nb10 mainly contributed to antigen binding via insertion into the shallow binding pocket of HA; similar roles are played by 19- and 24-residue regions of the broad-spectrum neutralizing antibodies CH65 and C05 (36, 37), respectively. The predicted structure suggests that Nb10 can be localized to the antigenic sites Sa, Sb, and Ca2 (38, 39), including the core conserved residues W153, K156, and L194 recognized by the antibody 13D4 (40), as well as Y98, W153, and E190 recognized by C12H5 (11). However, unlike C05 that neutralizes strains of the subtypes H1, H2, and H3, Nb10 cross-neutralizes a large subset of viruses of the H5 subtype but not those of other subtypes. This indicates that the constraints on the neutralization capacity of Nb10 for influenza viruses of the other subtypes may parallel those that impact the broader efficacy of CH65 (37). This prompted us to speculate that some of the binding regions of Nb10 may target the less-conserved residues around the RBS. For example, the conservation of residues D131, L131a, P145, D159, A188, and N193 is below 60% and ranges from 17.2% to 58.4% (Table S10).

Additionally, the antigenic epitopes recognized by other broad-spectrum neutralizing antibodies and which exhibit similarities with those recognized by Nb10 were analyzed herein. These two antibodies were isolated from individuals who recovered from H5 infection. The similar antigenic epitopes show that the antigenic epitope detected by Nb10 can not only induce an immune response in camels but also may enable infected humans to produce neutralizing antibodies against the epitope. As shown in Table S12, the numbers of amino acids in the antigenic sites recognized by FLD21.140 and AVFluIgG03 (18 and 19, respectively) are less than those recognized by Nb10. Previous studies have shown that FLD21.140 and AVFluIgG03, which display broad-spectrum neutralization capabilities against various viruses of the subtype H5, exhibit distinct complementarity in binding and neutralization, which may be attributable to their specificity and preferential recognition of residues at positions 133a, 144, and 145 of HA (41). However, we believe that Nb10 is highly probable to neutralize the current HPAIV of the subtype H5 from dairy cows, even though the critical residues in determining the sensitivity to Nb10 neutralization remain uncertain. First, the HA gene of the inactivated vaccine Re14 and the virus from dairy cows are both classified within the branch of 2.3.4.4b. The Nb10 exhibited a significant neutralizing effect against Re14/PR8, suggesting that Nb10 may also neutralize the H5 from dairy cows. Furthermore, we randomly acquired eight amino acid sequences of HA from infected dairy cows via the GISIAD website. Based on the binding epitopes of Re8 and Re14 predicted by AlphaFold 3, the residues of the interacted sites were compared with those at corresponding positions of the downloaded sequences, as illustrated in Table S13. All residues of the eight sequences demonstrate a high degree of consistency with Re14, except for one amino acid marked in red on a yellow background. Notably, the residues at position 192 of all downloaded sequences differ from Re14 but align with Re8. Consequently, the Nb10 may neutralize the HPAIVs of subtype H5 from dairy cows. Taken together, strains with single-site mutations based on the predicted interaction interface should be conducted to investigate the key antigenic sites recognized by Nb10 and to verify the accuracy of the predicted structure of the HA1:Nb10 complex. Additionally, the conformational epitope recognized by Nb10 may be further characterized via the isolation and analysis of escape mutants. These studies are expected to offer a theoretical foundation for the directed evolution of Nb10, the design and implementation of multivalent nanobodies, and the development of effective vaccines.

Most antibodies that target RBS exhibit high specificity for a particular subtype of the virus; examples include the antibodies 5J8, CH65, and 8M2 (37, 42–44). The exceptions that recognize RBS from more than one subtype of HA are few in number (36, 45, 46). Moreover, the heterosubtypic potency of certain antibodies appeared to hinge on multivalency for stronger binding (*K*_*D*_ in the nanomolar range) and broad-spectrum efficacy. As reported previously, the bivalent antibody S139/1 was considerably more effective in neutralizing the binding panel than the monovalent antibody S139/1 (47). Strikingly, the bivalent form of Nb10 displayed reduced IC_50_ values in HI and MN assays that employed the virus Re12/PR8 and showed neutralization potential against the virus Re5/PR8 in the HI assay (data not shown). These observations suggest that multivalency may enhance the cross-reactivity of Nb10 to beyond the viruses of clades 2.3.2.1 and 2.3.4.4. Therefore, an increase in avidity for the antigen is expected to enhance the spectrum of neutralization activity of nanobodies that target the RBS of HA1.

The delivery of antibodies directly into the lungs via the pulmonary route of administration has been proven to be effective for prevention and treatment (15, 18, 29). The pulmonary delivery of the antibodies CR9114, CR6261, and CF-404 via the intranasal and nebulization routes has been reported to be effective even at low doses of the antibodies (15, 48). Prophylactic treatments involving the nasal administration of H5-VHHb at a dose as low as 25 µg/kg resulted in reduced viral titers in the lungs that were below the limits of detection (27). The preventive benefits observed at low doses of the H5-VHHb are attributable to the delivery of the bivalent nanobody merely 4 h prior to infection against the challenge virus of a median lethal dose (MLD_50_) of barely 1. Consequently, the quantities of bivalent nanobodies with augmented affinity for the virus are likely to remain high for a prolonged duration post-exposure to the virus, which helps to inhibit viral replication (47). By contrast, the mice were subjected to a higher dose (10 MLD_50_) of infected virus, and the monovalent nanobody Nb10 was administered 24 h prior to the infection; the considerably earlier administration of Nb10 compared to the 4 h interval necessitates the employment of a higher concentration than that of the bivalent nanobody H5-VHHb.

Although the Y2H assay bypasses the limitations associated with phage display technology for antibody screening, including the necessity for extensive antigen purification and inadequate presentation of critical antigen sites, the bait and prey proteins produced in the Y2H system are devoid of glycosylation modifications (49). As a result, screening in yeast cells yielded nanobodies that were all directed against the non-glycosylated bait protein Re8-HA1. However, the HA, especially the region around RBS, is typically subjected to glycosylation. Previous studies have shown that the RBS region of HA from the strain Re8/PR8 contains an *N*-glycosylation site (50), unlike that of HA from the virus Re14/PR8 (data not shown). The occurrence of glycosylation modifications typically obscures the antigenic epitope, thereby hindering the recognition of the epitope by the antibody. Moreover, the RBS region is highly conserved. Therefore, the neutralizing effect of Nb10 against the recombinant virus Re14/PR8, which lacks glycosylation modifications in the RBS region, is slightly greater than that against virus Re8/PR8. The absence of glycosylation modifications may restrict the application of the Y2H technique for screening high-affinity nanobodies whose binding with the target proteins is dependent on post-translational glycosylation modifications. In addition, the results of ELISA revealed that only seven nanobodies exhibited strong binding affinities to inactivated Re8 virus out of a total of 23 nanobodies screened using yeast cells. This may be related to immunological antigen presentation or the absence of glycosylation of the target protein during the screening process. Antigens located in non-RBS areas may exhibit reduced immunogenicity compared to those in RBS regions. Moreover, nanobodies that target non-RBS regions may exhibit poor affinity for or fail to recognize glycosylated HA1. Furthermore, the protein HA1 was employed for Y2H screening, while HA is typically a trimeric protein. Several of the nanobodies obtained herein may, in fact, recognize the antigenic regions that are typically hidden in the trimeric protein.

The *in vivo* application of nanobodies for the protection of host organisms remains limited by many factors. First, intratracheal administration allows nanobodies to directly confer protection and facilitates neutralization of viruses via respiratory transfer. However, the 10-fold smaller size of nanobodies compared to that of the typical antibodies allows the easy and rapid penetration of these nanobodies into tissues, resulting in their rapid elimination from the bloodstream. Second, Nb10 primarily targets the highly mutable and important antigen HA1 of the H5 clades. Any alterations in the protein HA, particularly those that change critical antigenic epitopes in the RBS, can diminish the efficacy of the nanobody and facilitate antibody evasion. In addition, the utilization of nanobodies may impose selection pressure on the virus, leading to the emergence of escape variants. Therefore, an individual nanobody directed at specific antigenic epitopes typically fails to address escape variants, and numerous nanobodies are incapable of neutralizing viruses from divergent evolutionary lineages, as are most antibodies in other studies. Consequently, the application of multivalent or multivalent multispecific nanobodies, as also a nanobody cocktail, is expected to prove beneficial for *in vivo* therapy. Such an approach is expected to extend the serum half-life, boost its binding affinity, and improve the neutralization capacity of the nanobody against the virus, besides alleviating the risk of escape mutations (16, 47). Furthermore, the administration of nanobodies targeting various epitopes can prove effective against a broader range of viral strains. In addition, the fusion of the fragment crystallizable regions of immunoglobulin G (IgG) or immunoglobulin A with the nanobody enhances the efficiency of therapeutic nanobodies and improves their blood-circulation time (51–53). Further studies are required for validating the bioavailability, distribution, and half-life of multivalent multispecific nanobodies and the protective efficacy of the cocktail therapy.

## MATERIALS AND METHODS

### Cells, viruses, and proteins

Human embryonic kidney 293T (HEK293-T) and Madin–Darby canine kidney (MDCK) cells were obtained from the American Type Culture Collection and cultured in Dulbecco’s Modified Eagle’s Medium (complete DMEM; Gibco, USA) supplemented with 10% fetal bovine serum (FBS, Gibco) and an antibiotic solution consisting of penicillin and streptomycin (Gibco) at 37°C in an incubator that maintains a carbon dioxide (CO_2_) concentration of 5%. Sf9 cells were maintained in suspension in serum-free medium (Gibco) at 28°C under rotary conditions (120–130 rpm). All the cell lines were tested and found to be negative for mycoplasma contamination.

The recombinant H5 AIVs (a 7:1 reassortant virus containing seven segments from the A/Puerto Rico/8/1934 [H1N1] virus and HA segment from the commercially available inactivated vaccine strains) were constructed by reverse genetics using the PR8 backbone and a previously described protocol (50, 54). Briefly, eight internal gene segments of the influenza virus were inserted into the plasmid pDZ at *Bsm*BI restriction enzyme sites, respectively. Moreover, the eight plasmids so constructed were employed for the cotransfection of HEK293-T cells using Lipo2000 (Invitrogen, USA) to obtain the first generation of rescued virus. The recombinant viruses (shown in Table S3) were utilized for evaluating the neutralization activities of the nanobodies, as well as for the investigation of underlying mechanisms and assessments of the preventive and therapeutic activities of Nb10. These recombinant viruses were propagated in 10-day-old embryonated specific-pathogen-free (SPF) chicken eggs. The viral titers were determined by the Reed–Muench method using a standard median tissue culture infectious dose at 50% (TCID_50_) assay, as described previously (55, 56). The reassortant H5 viruses so obtained were labeled with PR8 in the current study; for instance, Re6/PR8 bears the gene encoding HA (synthesized by General Biosystems, China) of A/duck/Guangdong/S1322/2010, which is the HA gene in the Re6 inactivated vaccine.

The recombinant HA1 domain (residues 11–329; numbering corresponds to the HA sequence from the H3 virus; Table S1) was generated using a baculovirus expression system and the baculovirus insect cell line Sf9, as described previously (57). Briefly, the gene encoding the HA1 domain, along with a secretion signal and an 8x-His-tag at the C-terminus, was amplified and ligated with linearized baculovirus shuttle vector pFastBac Dual using an infusion cloning Kit (Takara Bio USA Inc., Japan). The recombinant bacmid so obtained was employed for the transfection and propagation in Sf9 cells according to the instructions provided in the user handbook of the Bac-to-Bac Baculovirus Expression System (Invitrogen). After the infection of cells with the recombinant baculovirus for 3 days, the protein HA1 was expressed in the culture supernatant and recovered using Ni-NTA agarose (GenScript, China) as per the manufacturer’s instructions. The concentration of the protein so obtained was determined using the BCA Protein Quantification Kit (Vazyme, China) as per standard procedure. The recombinant HA1 domain so obtained from the baculovirus insect cell line (listed in Table S4) was used for binding analyses, including ELISA and SPR. All the primers used in the current study are listed in Table S5. The plasmids and strains employed herein are available in the supplemental material, specifically in Tables S6 and S7. In this research, ReX-HA1 refers to the amino acid sequence of HA in the recombinant protein that originated from the ReX inactivated vaccine strain. For example, Re8-HA1 signifies that the amino acid sequence of HA1 in the recombinant protein is derived from the Re8 strain of the inactivated vaccine.

### Construction of the Y2H library

A healthy 2-year-old male Bactrian camel was immunized with 3 mL of the inactivated viral vaccine H5-Re8 (Harbin Weike Biotechnology Development Company, China). The procedure was performed five times in total with 3 week intervals. Three weeks following the administration of the last booster dose, 200 mL of blood was drawn for the isolation of PBMCs. Density gradient separation using Ficoll-Hypaque gradient (GE Healthcare, USA) was employed for isolating the PBMCs. Total RNA was extracted from the PBMCs using TRIzol (Thermo Fisher, USA) according to the manufacturer’s instructions. The gene fragments that encode VHH were amplified by two-step nested PCR. Reverse transcription PCR was employed for amplifying the VH–CH1–CH2 and VHH–CH2 regions using the forward and reverse primers CALL001 and CALL002, respectively, and SuperScript III First-Strand Synthesis System (Invitrogen) (58). The amplified fragments of approximately 750 bp size were purified using the QIAquick Gel Extraction Kit (Qiagen, Germany) and employed as the template for the second PCR. The VHH coding sequences of approximately 400 bp size were amplified via the second PCR using the nested primers VHH-forward and VHH-reverse. The subsequent step involved the ligation of the VHH cDNAs with the linearized vector pGADT7-Rec, which encodes the prey proteins fused with the activation domain of Gal4. The plasmid was then introduced into competent cells of yeast Y187 (Coolaber, China) via the lithium acetate transformation technique. The Y2H library was then obtained as per the instructions specified in the Make Your Own “Mate & Plate” Library System User Manual (Clontech, USA). A 10 µL aliquot of the library was diluted (1:100, 1:1,000, 1:10,000, and 1:100,000) and spread onto 100 mm plates containing synthetic dropout (SD) medium without leucine (SD/−Leu) agar. The plates were incubated at 30°C for 72–96 h, and the number of independent clones was counted to estimate the size of the constructed Y2H library. Ten unique clones were randomly selected for PCR amplification to evaluate the rate of recombination in the library using the universal primers GAL4AD-F and 3AD-R. The amplified fragments were then sequenced to evaluate library diversity. All the primers employed in this study are listed in Table S5. These Y2H libraries were stored at −80°C in 1 mL aliquots.

### Y2H screening

Y2H screening is a crucial method for studying protein–protein interactions and was employed herein for the identification of nanobodies that recognize HA1. The coding sequences of the bait protein Re8-HA1 were amplified using the primers bait-HA1-F and bait-HA1-R and ligated with linearized pGBKT7. The bait protein was expressed as a fusion protein containing the yeast Gal4 DNA-binding domain. The recombinant vector was employed for the transformation of chemically competent cells of the bait strain Y2HGold (Coolaber), as specified for the Yeastmaker Yeast Transformation System 2 (Clontech). The cells were then plated on SD medium lacking tryptophan (SD/−Trp; Takara Bio USA, USA) agar. The Y2HGold strain containing the plasmid pGBKT7-HA1 was assessed for the absence of autoactivity and toxicity, and the bait strain was then employed for screening. For the screening process, the freshly cultured bait strain was concentrated and mixed with 1 mL of prey library strain Y187 (pGADT7-preys), as per the instructions in the user guide for the Matchmaker Gold Yeast Two-Hybrid procedure (Takara Bio USA). The strains were then allowed to mate for 20 h. As a control for the Y2H screening, the Y2HGold strain was cotransformed with the bait plasmid and pGADT7-empty. The cells were then spread onto DDO/X/A [SD/−Leu/−Trp supplemented with 40 µg/mL X-alpha (α)-Gal and 200 ng/mL aureobasidin A, Takara Bio USA] plates and incubated at 30°C for 3–5 days, resulting in the formation of blue colonies. The blue colonies that thrived on the DDO/X/A plates were transferred onto QDO/X/A agar plates of increased stringency. The QDO/X/A plates lacking adenine, histidine, tryptophan, and leucine were supplemented with X-α-Gal and aureobasidin A. The potential positive clones so derived were subjected to DNA sequencing. The Y2HGold strain was subsequently cotransformed with the positive plasmid pGADT7-preys along with either pGBKT7-HA1 or pGBKT7-empty to distinguish genuinely positive interactions from the false-positive ones. Simultaneously, the Y2HGold strain carrying the plasmids (pGBKT7-53 or pGBKT7-Lam) was mated with the strain Y187 harboring the plasmid pGADT7-T as positive or negative control (reference) strains, respectively. The maximum likelihood method and MUSCLE were employed for the construction of a phylogenetic tree based on the amino acid sequences of nanobodies using the software MEGA11 (version 11.0.13).

### Production and characterization of nanobodies

Full-length VHH sequences containing either the 6x-His-tag or Twin-Strep-tag at the C-termini were obtained from General Biosystems. These VHH sequences were then cloned into the expression vector pPICZα A at the *Eco*RI and *Xba*I sites, which was confirmed by PCR using the primers 5α-Factor-F and 3AOX1-R. As per the manufacturer’s instructions (Invitrogen), the yeast strain *P. pastoris* X33 (Mut^+^ and His^+^) bearing the recombinant plasmids was cultivated in yeast extract peptone dextrose medium containing 100 µg/mL zeocin. The strain X33 harboring the constructs of interest was cultured in buffered minimal glycerol medium to promote growth during the log phase and then transferred to buffered minimal medium containing 1% (vol/vol) methanol; the culture was then incubated at a temperature of 28°C–30°C under rotary conditions (250–300 rpm) to induce the expression of the nanobodies. After 3 days, the culture supernatant was harvested and purified.

### ELISA

The binding capacities of all the selected nanobodies were evaluated using indirect ELISA. The inactivated virus H5-Re8 (20 µL) suspended in carbonate–bicarbonate coating buffer (100 µL/well, 0.35 M NaHCO_3_, 0.15 M Na_2_CO_3_, and pH 9.6) was coated onto high-capacity binding plates (Jet Bio-Filtration, China) and incubated overnight at 4°C. The plates were blocked with 3% bovine serum albumin (BSA). The VHHs containing the 6x-His-tag (10 µg/mL) were subjected to twofold serial dilutions and then added to the wells, followed by incubation for 1 h at room temperature. The bound nanobodies were then identified using anti-his-tag mouse mAb (GenScript) and horse radish peroxidase (HRP)-labeled goat antimouse IgG (Sangon, China).

The EC_50_ values, which represent the concentration at which 50% of the maximal effect is achieved, were calculated for assessing the binding affinities of the nanobodies to HA1 using ELISA (59). Following the coating of HA1 (1 µg), twofold serial dilutions of the VHHs containing Twin-Strep-tag (initial concentration of 10 µg/mL) were added to the wells and incubated for 1 h. Subsequently, 100 µL of anti-Strep Tag II mouse mAb (1:2,000 dilution; GenScript) and HRP-labeled goat antimouse IgG (1:5,000 dilution) were added sequentially followed by incubation for 1 h each time, finally followed by color development using 3,3′,5,5′-tetramethylbenzidine. The absorbance at 450 nm was measured using a plate reader (Bio-Tek Epoch 2; Agilent Technologies, USA) after the reaction was halted with 2 M sulfuric acid. The plates were subjected to three washes with PBS containing Tween-20 (PBST, 0.05% vol/vol) after each of the preceding processes. All the samples were analyzed in triplicate. The EC_50_ values were calculated by employing non-linear regression analysis using the software GraphPad Prism (version 9).

The ELISA carried out for epitope identification involved the coating of an excess of Nb10 containing the 6x-His-tag overnight on the plate. To identify whether steric hindrance by Nb10 affected the interaction between the other identified nanobodies and HA1, protein HA1 (1 µg) was added to the wells before the application of Nb10, Nb20, Nb30, Nb42, Nb45, and Nb52, all containing the Twin-Strep-tag. Twofold serial dilutions of these nanobodies, beginning with an initial concentration of 10 µg/mL, were employed for the assay. The measurement was performed in triplicate, as described previously for the estimation of binding affinity using ELISA.

### HI assay

The HI assay was used for identifying whether the nanobodies were capable of inhibiting hemagglutination. As previously reported, the HA titers of the recombinant virus were first determined using a standard hemagglutination assay with 1% chicken red blood cells (RBCs) in the absence of antibodies (60). For the HI assay, the purified VHHs were subjected to twofold serial dilution in PBS in 96-well V-bottom plates and combined with an equal volume of four hemagglutinin units for 40 min at room temperature. Each well was supplemented with an equivalent quantity of 1% chicken RBCs, and the mixture was gently agitated and incubated for 30 min (61). The HI titer was determined by button formation, and IC_50_ was defined as the nanobody concentration that inhibited hemagglutination in 50% of the RBCs (62). All the VHHs were assessed via duplicate measurements.

### WB analysis

WB analysis was conducted for precisely investigating the type of epitopes on HA1 (59). The purified Re8-HA1 proteins, as well as the nanobody containing the Twin-Strep-tag (employed as the control), were separated on 12% SDS-PAGE gels and then electroblotted onto 0.45 µm polyvinylidene difluoride membranes (Millipore, USA). The membranes were blocked with 5% non-fat milk and subsequently immersed in Nb10 containing the Twin-Strep-tag; this was followed by incubation with anti-Strep Tag II mouse mAb. The other blot containing only HA1 was probed with antihis-tag mouse mAb. Following washes with PBST, the presence of the bound antibody was determined by incubating the membrane with HRP-conjugated goat anti-mouse IgG, washing with PBST, and finally, visualization using enhanced chemiluminescence Western blotting substrate (Yeason, China).

### MN assay

A cell-based MN assay was conducted for assessing the neutralizing capacity of the nanobodies against viruses of subtype H5 under *in vitro* conditions, as previously reported (61, 63). In summary, MDCK cells were seeded onto 96-well cell culture plates and allowed to adhere overnight to form a monolayer of cells. Twofold serial dilutions of VHHs were mixed with an equal volume of viral suspension corresponding to 100 TCID_50_ of the H5 recombinant viruses of different clades and incubated for 1 h at room temperature; the virus–nanobody mixtures were then transferred onto the preseeded MDCK cells followed by further incubation for an hour. The cells were then washed with PBS, followed by the addition of fresh minimum essential medium containing 2% FBS and the antibody at the appropriate concentration and cultured for 18–22 h at 37°C in the presence of 5% CO_2_. The cells were then rinsed, fixed with 80% acetone for 90 min at −20°C, and then blocked with 3% BSA. Subsequently, the infected cells were detected using influenza A mouse anti-NP mAb (GenTex, USA), which is specific for the NP protein of the influenza virus. The cells were visualized following staining with antimouse IgG fluorescein isothiocyanate (FITC, Sangon). Neutralization endpoints corresponded to a reduction in viral infection to 50% of the levels obtained with the control treatment using IFA, with the corresponding titers of VHHs representing the IC_50_ values. Moreover, negative controls containing only cells and only viruses were also included in the assay. The assay was performed using triplicate samples.

### Cell-based assay for the inhibition of viral entry

A cell-based assay using IFA for evaluating the inhibition of viral entry was designed to elucidate whether the nanobodies functioned at an early stage of viral infection (62, 64). A mixture containing the nanobody (5 or 20 µg/mL) and the virus Re8/PR8 at a multiplicity of infection (MOI) of 10 was incubated for an hour at 37°C. The mixture was then added to MDCK cells and incubated for 1 h at 4°C, followed by washing with PBS to remove unbound virions. The cells were then replenished with DMEM enriched with 2% FBS and containing the nanobody at the indicated concentrations. Following incubation for 16–18 h at 37°C under conditions of 5% CO_2_, the cells were fixed with polyoxymethylene and permeabilized with 0.2% Triton X-100. Subsequently, influenza A mouse anti-NP mAb and FITC-labeled antimouse IgG polyclonal antibody were used for probing the infected cells, and 4′,6-diamidino-2-phenylindole was employed for labeling the nuclei of cells. Immunofluorescence was observed using an inverted fluorescence microscope (Ti2-E; Nikon, Japan).

### Assay for the inhibition of syncytia formation

To further assess whether the nanobody inhibits membrane fusion, a cell–cell fusion experiment was conducted as previously reported, with a few minor adjustments (65, 66). Monolayers of MDCK cells established in six-well plates were infected with virus Re8/PR8 at an MOI of 0.5 for 22 h and then replenished with DMEM containing 2% FBS. The culture supernatants were subsequently removed and replaced with DMEM containing the nanobody (5 or 20 µg/mL), followed by incubation for an hour. The cells were then treated with citric acid (pH 5.5) at 37°C for 10 min, which was subsequently replaced with DMEM containing 2% FBS. Following a 3 h incubation, the cells were washed and fixed with 4% polyoxymethylene. Finally, syncytium formation was observed by applying Giemsa stain as per the manufacturer’s instructions, and photomicrographs were obtained.

### Egress inhibition assay

Egress inhibition assay was undertaken to investigate the potential involvement of nanobodies in inhibiting viral release (11, 67). MDCK cells were infected with virus Re8/PR8 at an MOI of 2 for 4 h in maintenance medium (DMEM supplemented with 2% FBS). After 4 h, the culture supernatants were discarded, followed by three washes with PBS for removing any non-internalized virus. The cells were then treated with the nanobody in a maintenance medium and cultured for 18–22 h. Subsequently, the culture supernatants and cell lysates were collected for evaluating the viral particles using WB; influenza A mouse anti-NP mAb and HRP-conjugated secondary antibody were employed for detecting the virus.

### SPR analysis

The binding kinetics and affinities of the nanobodies toward HA1 were evaluated herein by monitoring the rates of association (association rate constant [*k*_*a*_], M^−1^s^−1^) and dissociation (dissociation rate constant [*k*_*d*_], s^−1^) at 25°C by SPR analysis using Biacore 8K (GE Healthcare). The purified his-tagged HA1 protein in PBS buffer was loaded onto the Ni-NTA sensor chip at a concentration of 10 µg/mL. The flow cell one without immobilized HA1 served as the reference signal from non-specific binding and bulk refractive index effects. For the kinetics measurements, the ligand-coated biosensor was exposed to different concentrations of the analytes (twofold serial dilutions ranging from 2,000 to 125 nM) delivered at 30 µL/min for a 120 s association phase and a subsequent 600 s dissociation phase, followed by surface regeneration with the regeneration solution (350 mM ethylene diamine in PBS). The raw data were obtained upon subtracting the background binding signal corresponding to the reference flow cells. The raw data corresponding to association and dissociation were globally fitted with a 1:1 (Langmuir) binding model using the multicycle method of Biacore Insight Evaluation Software (GE Healthcare). *K*_*D*_ was derived from the ratio of *k*_*d*_ to *k*_*a*_. The binding affinity curves were processed using GraphPad Prism software.

### Studies on the prophylactic and therapeutic efficacy of Nb10 in mice

Owing to the significant cross-neutralizing activity of Nb10 against the viruses of subtype H5, viruses Re6/PR8, Re8/PR8, Re10/PR8, Re11/PR8, and Re14/PR8 were selected for evaluating the preventive and therapeutic efficacy of Nb10 in a BALB/c mouse challenge model. Female BALB/c mice aged 6 weeks were obtained from SPF Biotechnology Co., Ltd. (China) and randomly assigned to prophylactic and therapeutic efficacy groups. As described previously, the MLD_50_ assay was performed by first anesthetizing mice with inhaled isoflurane (3%) followed by intranasal challenge with various recombinant viruses. The mice were then administered a mixture of ketamine (75 mg/kg) and medetomidine (1 mg/kg) via the intraperitoneal route. Either prior to or following the intranasal infection with the recombinant virus, the mice were administered (via the intratracheal route) different doses of the nanobody using a microsprayer aerosolizer (Yuyan Bio, China) (18). To evaluate the preventive efficacy of the nanobody, groups of eight mice were administered Nb10 (2 or 3 mg/kg) a day before the intranasal inoculation with 10 MLD_50_ of the recombinant virus. To investigate the therapeutic efficacy of Nb10, groups of eight mice were administered at doses of 2.5 or 3.5 mg/kg for the Re8/PR8 as well as Re14/PR8 groups and 4 or 8 mg/kg for the Re6/PR8, Re10/PR8, and Re11/PR8 groups 24 h post-infection, as well as 3.0 or 4.5 mg/kg for the Re14/PR8 group 24 or 48 h after infection. Similarly, infected mice were treated with PBS as a control. The changes in body weight and mortality were monitored daily from the day of infection with the influenza virus (day 0) until day 14. The mice that experienced a loss of over 25% of their initial weight (corresponding to day 0) were defined as non-surviving (humane endpoint). Three mice from each group were euthanized to assess viral loads in the right lungs using the classic TCID_50_ assay. The data on weight loss, survival, and viral titers were analyzed using GraphPad Prism.

### Analysis of viral titers and histology of the lung

For the evaluation of pulmonary titers, the right lungs were removed aseptically, homogenized in PBS, and stored at −80°C. Prior to application onto confluent monolayers of MDCK cells in 96-well cell culture plates, the homogenates were thawed and centrifuged at 10,000 × *g* at a temperature of 4°C. Next, 10-fold serial dilutions of the supernatant samples in DMEM were added to the wells of the plates seeded with MDCK cells in a total volume of 100 µL and incubated at 37°C for 1 h under conditions of 5% CO_2_. The cells were subsequently cultured for 2 days followed by hemagglutination assay. The TCID_50_ values were visualized and quantified by button formation of the hemagglutination test following the Reed–Muench method. The remaining lung halves were collected for histology analysis, as described previously. The lung tissue was fixed with 10% neutral buffered formalin for at least 1 day, followed by dehydration with ethanol and embedding in paraffin. The lung tissue was then sliced to yield thin slices of typically 5–8 µm; the slices were affixed to glass slides and subjected to staining with hematoxylin and eosin. The severity of the histologic lesions was examined and scored based on the percentage area that exhibited histopathological abnormalities such as hemorrhage, alveolar and interstitial edema, alveolar wall thickening, and suppurative inflammation that included the infiltration of polymorphonuclear leukocytes and/or mononuclear cells (lymphocytes, plasma cells, and macrophages). The following scoring system was employed: 0, no lesions; 1, minimal lesions (1%–20%); 2, moderate lesions (21%–50%); and 3, severe lesions (51%–100%).

### Epitope mapping and structure prediction

Yeast display has emerged as a promising technique in the field of protein engineering and has been employed herein as a platform for the expression of various antigenic peptides and full-length proteins. Sequences corresponding to the truncated fragments of HA1 and the full-length protein were cloned into the *Hin*dIII and *Nhe*I restriction enzyme sites of the pYD1 vector (Invitrogen) and amplified in competent cells of *Escherichia coli* DH5α. *S. cerevisiae* EBY100 (Coolaber) was transformed with each of the constructed plasmids; the cells were then streaked on plates containing SD-CAA (SD/−Trp medium, tryptophan auxotrophic, 2% D-glucose, 1 M sorbitol, pH 6.40, and 25 µg/mL kanamycin; Coolaber) agar and incubated at 30°C for 2–4 days. The transformants analyzed by colony PCR were grown in SD/−Trp medium overnight, transferred to a medium containing galactose (SC/−Trp with 2% D-galactose) for the induction of expression of HA1 and its fragments, and incubated at 30°C under rotary conditions (250 rpm) for 48–72 h. Subsequently, the cells were centrifuged, treated with 3% BSA, and then exposed to Nb10 labeled with the Strep tag. This was followed by the addition of anti-Strep Tag II mouse monoclonal antibody and detection using FITC-labeled goat antimouse IgG. Immunofluorescence was visualized using a fluorescent microscope, and images of the samples were captured. Furthermore, the amino acid sequences of Re8-HA1 and Nb10, Nb20, Nb30, Nb42, Nb45, and Nb52 or Re14-HA1 and Nb58 were individually subjected to analysis using AlphaFold 3 (https://alphafoldserver.com/) to obtain a 3D model structure of the antigen–nanobody complex. The protein–protein interface was further analyzed using the online tool PDBePISA (https://www.ebi.ac.uk/pdbe/pisa/). The PyMOL software was utilized for visualizing the residues involved in the interaction between the nanobodies and antigens.

### Statistical analysis

GraphPad Prism (version 9) software was used for data analysis. Statistical significance between survival curves was analyzed using Kaplan–Meier survival analysis with a log-rank test. The results were analyzed using one-way or two-way analysis of variance as stated in the figure legends. A *P* value of <0.05 was considered statistically significant. Asterisks indicate the statistical significance: ns, no significance; *P < 0.05, **P < 0.01, ***P < 0.001, ****P < 0.0001.

## Data Availability

All data generated or analyzed during this study are included in this published article and its supplemental material.

## SUPPLEMENTAL MATERIAL

Supplemental material jvi.02090-24-s0001.doc Tables S1 to S4 and Figures S1 to S4.

ASM does not own the copyrights to Supplemental Material that may be linked to, or accessed through, an article. The authors have granted ASM a non-exclusive, world-wide license to publish the Supplemental Material files. Please contact the corresponding author directly for reuse.
