# Supplementary material for: Identification and characterization of a broadly neutralizing and protective nanobody against the HA1 domain of H5 avian influenza virus hemagglutinin
Source: J Virol. 2025 Apr 7;99(5):e02090-24. doi: 10.1128/jvi.02090-24 (PMC12090751; doi:10.1128/jvi.02090-24)
Supplement: Supplemental material — Tables S1 to S4 and Figures S1 to S4. [file jvi.02090-24-s0001.doc]

**Supplemental material for**

**Identification and characterization of a broadly neutralizing and protective nanobody against the HA1 domain of H5 avian influenza virus hemagglutinin**

Siqi Xu,^a^ Yutong Liu,^a^ Chenying Luo,^b^ Mengruo Zhou,^a^ Ke Wang,^a^ Qianmei Xie,^a^ Qi Zhang,^a^ Qinying Zhang,^a^ Qianyu Li,^a^ Zhichao Pan,^a^ Saixiang Feng,^a^# Ming Liao,^a,c,d^#

^a^College of Veterinary Medicine, South China Agricultural University, Guangzhou, 510642, China

^b^College of Life Sciences, South China Agricultural University, Guangzhou, 510642, China

^c^Institute of Animal Health, Guangdong Academy of Agricultural Sciences, Guangzhou, 510640, China

^d^Zhongkai University of Agricultural and Engineering, Guangzhou, 510550, China

**#Address correspondence:**

Saixiang Feng: +86-20-85280718; fengsx@scau.edu.cn

Ming Liao: +86-20-89003055; mliao@scau.edu.cn

**Contents**

**Table S1. Nucleotide sequences of nanobodies and Re8-HA1.**

**Table S2. The Characterization of the neutralization concentration.**

**Table S3. Recombinant virus strains used in this study.**

**Table S4. Recombinant proteins used in this study.**

**Table S5. Primers used in this study.**

**Table S6. Plasmid used in this study.**

**Table S7. Bacterial and yeast strains used in this study.**

**Table S8. Interface identification Re8-HA1:Nb10 by PISA Program.**

**Table S9. The confidence and binding bonds of HA1: nanobodies protein complex.**

**Table S10. Sequence conservation of nanobodies epitopes in all H5.**

**Table S11. Interaction analysis of Re8-HA1:Nb10 by PISA Program**

**Table S12. The comparison of antigenic epitopes.**

**Table S13. The comparison of residues between the interacted sites of Re8 and Re14 recognized by Nb10 and the download sequences.**

**Fig. S1 Presentation of the selecting procedure and nanobody expression.**

**Fig. S2 Binding characteristics of neutralizing nanobodies.**

**Fig. S3 SDS-PAGE analysis of expression of the HA1s.**

**Fig. S4 Structures analysis of nanobodies separately binding to HA1 by AlphaFold 3.**

**Table S1. Nucleotide sequences of nanobodies and Re8-HA1.**

| **Name** | **DNA sequence (5'–3')** |
| --- | --- |
| Nb02 | CAGGTGCAGCTGGTGGAGTCTGGGGGAGGCTCGGTGCAGGCTGGAGGGTCTCTGAGACTCTCCTGTGCAGCCTCTGGATACACCTTTAGTAGTAATTGTATGGCCTGGTTCCGCCAGGCTCCAGGGAAGGAGCGCGAGGGGGTCGCAGTTATTTATACTCGTAGTATAAATAGCGCATACTATTCCGACTCCGTGAAGGGCCGATTCACCATCTCCCAAGACAAGGCCAAGAACACGGTGTATCTCCAAATGAACAGCCTGAATCCTGAGGACACTGCCATGTACTACTGTGCGGCAGATTCGGTCTGGCGCGGTGGTTACTGCGGCACCGCCACCCGTAGTTATAACTACTGGGGCCAGGGGACCCAGGTCACCGTCTCCTCA |
| Nb06 | CAGGTGCAGCTGGTGGAGTCTGGGGGAGGCTTGGTGCAGCCTGGGGGGTCTCTGAGACTCTCCTGTGCAGCCTCTGGATTCACCTTCAGTAACAACTGGATGCACTGGGTCCGCCAGGCCCCAGGGAAGGGGCTGGAGTGGGTGTCCAGTATCAATACTGGGGATGGTAGCACAAACTCTGCAGACTCCGTGAAGGACCGATTCACCATCTCCAGAGACAATGCCAAGAACACGCTGTATCTGCAATTGAACAGCCTGAAAACTGAGGACACGGCCATGTATTACTGTGCAAAAGCGGCCGCCTCGTGGCATATTATTCATTCCTGGGGCCAGGGGACCCAGGTCACCGTCTCCTCA |
| Nb07 | CAGGTGCAGCTGGTGGAGTCTGGGGGAGGCTTGGTGCAGCCTGGGGGGTCTCTGAGACTCTCCTGTGCAGCCTCTGGATTCACCTTCAGTGCCTACTACATGACCTGGGTCCGCCAGGTTCCAGGGAAGGGGCTGGAGTGGGTGTCCAGTATTTATCGTGATGGTAGTAACACATACTATGCAGACTCCGTGAAGGGCCGATTCACCATCTCCAGAGACAACGCCAAGAACACGGTGTATCTGCAAATGAACAGCCTGAAATCTGAGGACACGGCCCTGTATTACTGTGCCGCTGACAGGACGGTGGTTGAATACAGGTATCTCGAAGTTTGGGGCCAGGGCACCCAGGTCACCGTCTCCTCA |
| Nb10 | CAAGTTCAGTTACAGGAATCTGGGGGAGGCTCGGTGCAGGCTGGAGGGTCTCTGAGACTCTCCTGTTCAGCCTCTGAATACACTCGAAGTATGGCCTGGTTCCGCCAGGTTCCAGGGAAGGAGCGCGAGGGGGTCGCAGCTATCGATACTGGTAATGGGAACACATACTATCCCACCGTAGACGGCCGATTCATCATCTCCCGAGGCAACGCCAAGAACTCCGTAGATCTGGAAATGAACAGCCTGACACCTGACGACACTGCCATCTACTACTGTGCGGCTACGCAGGGCCCCCTCTGGCCTACTTTAGGGACTCAGTTTTCGACTGAAAGTTATAATTACTGGGGCCAGGGGACCCTGGTCACCGTCTCCTCA |
| Nb11 | CAGGTGCAGCTGGTGGAGTCTGGGGGAGGCTCGGTGCAGGCTGGAGGGTCTCTGAGACTCTCCTGTGCAGCCTCAGGATACATCCACAGTGGCTACTGCATGGGCTGGTTCCGCCAGGCTCCAGGGAAGGAGCGCGAGGGGGTCGCAGGTATTGATAGTGATGGTAGGATAAGCTACGCAGACTCCGTGAAGGGCCGATTCGCCATCTCCCAAGTGAACGCCAAGAACACTCTGCTTCTGCAAATGAACAGCCTGAAACCTGAGGACACTGCCATGTACTACTGTGCGGCAGGACGAGTATGTGACCTAATGTACATTCTGGGTTATAATCACTGGGGCCAGGGGACCCAGGTCACCGTCTCCTCA |
| Nb12 | CAGGTGCAGCTGGTGGAGTCTGGGGGAGGCTTAGTGCAGCCTGGGGGGTCTCTGAGACTCTCCTGTGCAGCCTCTGGATTCACCTTCAGTAGCTACTACATAGGCTGGGTCCGCCAGGCTCCAGGGAAGGGGCTGGAGTGGGTGTCCAGTATTTATAGTGATGGTAGTAATACATACTATGCAGCCTCCGTGAAGGGCCGATTCACCATCTCCAGAGACAACGCCAAGAACACGCTGTATCTGCAAATGAACAGCCTGAAAACTGAGGACACTGCCGTGTATTACTGCGCCACACGGTCGCGGTACTGGGGCCAGGGGACCCAGGTCACCGTCTCCTCA |
| Nb14 | CAGGTGCAGCTGGTGGAGTCTGGGGGAGGCTTGGTGCAGCCTGGGGGGTCTCTGAGACTCTCCTGTGCAGCCTCTGGATTCACATTCAGTATATACGACATGAGCTGGGTCCGTCAGGCTCCAGGGAAGGGGCTCGAGTGGGTCTCAGCTATGAATAGTGGCGGTAGTAGCACATATTATGCAGACTCCGTGAAGGGCCGATTCACCATCTCCAGAGACAACGCCAAGAACACGCTGTCGCTGCAAATGAACAGCCTGAAAACTGAGGACACTGGCGTGTATTATTGCACCACAGGCGCGGGCCGTAGTTGGTACTCAAAGGACTGGGGCCAGGGGACCCAGGTCACCGTCTCCTCA |
| Nb16 | CAGGTGCAGCTGGTGGAGTCTGGGGGAGAATCGGTGCAGGCTGGAGGGTCTCTGAGCCTCTCCTGCTCAGCCTCTGGAGAGTGCATGGGCTGGTTCCGAGAGGCTCCAGGAAAGGAGCGCGAGGGGGTCGCACTTATGCATAGTGATGGTGCCACAACCTATGGAGACTCCGTGAAGGGCCGATTCACCATCTCCCATGACAACGCCAAGAACACTCTGTATCTCGACATGAACAGCCTGAAACCTGAAGACACTGCCATGTACTACTGTGCGGCAGACATAGCAGTTTGTGATATGCGTTGGGTCGTCACATCTGACTTTGGTCCATGGGGCCCGGGGACCCAGGTCACCGTCTCCTCA |
| Nb17 | CAGGTGCAGCTGGTGGAGTCTGGGGGAGGCTTGGTGCAGCCTGGGGGGTCTCTGAGACTCTCCTGTGCAGCCTCTGGATTCACCTTCAGTAACAACTGGATGCACTGGGTCCGCCAGGCCCCAGGGAAGGGGCTGGAGTGGCTGTCCAGTATTTATACTAGTGATGGTAACACAAACTCTGCAAACTCCGTGAAGGGCCGATTCACCATCTCCAGAGACAACACCAACAACATGCTGTCCCTGCAAATGAACAGCCTGAAATCTGAGGACACGGCCCTGTATTACTGTATCAGGTCGGACTATGGGTTGGGTACCATGACGACCGACTGGGGCCAGGGGACCCAGGTCACCGTCTCCTCA |
| Nb20 | CAAGTTCAGTTACAGGAATCTGGGGGAGGCTTGGTGCAGCCTGGGGGGTCTCTGAGGCTCGCCTGCGCAGCATCAGGATTCACCTTACAGAGCGACTCCATCGCCTGGGTCCGCCTGGCTCCAGGGAAGGGCCTGGAGTGGGTGTCCAGCATTTATAGTAATAGTCACAACACATTCTATGCACAGTCCGTGATGGGCCGATTCACCATCTCCAGAGACTTCGCCAAGGACACGACGTATCTGCAAATGGACAATTTGAAATCTGAAGACACGGCCCTGTATTTTTGTGCCGCTGATCCCCGAATCAGTCTCCCCGATTTGCTGGTAGCTGGCACGGTCTCACTCGCTGACTTTGGTTATTGGGGCCAGGGGACCCAGGTCACCGTCTCCTCA |
| Nb24 | CAGGTGCAGCTGGTGGAGTCTGGGGGAGGATCGGTGCAGGCTGGAGGGTCTCTGAGACTCTCCTGTGTAGCCTCGTCATACACCGTCAGTAACTACTGCATGGGCTGGTTCCGCCAGGCTCCAGGAAAGGAGCGCGAGGGGCTCGCATCTATTGATAGCAATGGTAGGACGGTGTACGCAGACTCTGCGAAGGGCCGATTCACCATCTCCGTAGACAACGCCAAATACACTCTGTATCTCCAAATGAGCAGCCTGAAACCTGACGACACTGCTATGTACTACTGTGCGGTAGATCTTTGGAACCATGACTGCGTTGCAGAGTCTCGGCGGTCTTTTGGTTACTGGGGCCAGGGGACCCAGGTCACCGTCTCCTCA |
| Nb25 | CAGGTGCAGCTGGTGGAGTCTGGGGGAGGCTTGGTGCAGCCTGGGGGGTCTCTGACACTCTCCTGTGAAGCCTCTGGATTCACCCTCAGTAACTATTACATGGACTGGGTCCGCCAGGCTCCCGGGAAGGGACTCGAGTGGGTCTCAACTATTCATGGTGGTGCCACATACTATGCAGACTCCGTGAAGGGCCGATTCACTATCTCCAGAGACAACGCCAAGAACACTCTATATCTACAATTGAACAACCTGAAAACTGAGGACACGGCCATGTATTACTGTACAAAACTGGGATCCTTATTACTAGTGGTCGCAGGGTGGGGCCAGGGGACCCAGGTCACCGTCTCCTCA |
| Nb28 | CAAGTTCAGTTACAGGAGTCTGGGGGAGCCTTGGTGCAGCCTGGGGGGTCTCTGAGACTCTCCTGTGCAGCCTCTGGATTCACCCTCAGTACTTATGGGATGTACTGGGTCCGCCAGACTCCAGGGAAGGGACTCGAGTGGGTCTCGTTTATTAATGGTGGTAGTGGTAGCACATTCTATCCAGACTCCGTGAAGGGCCGATTCACCATCTCCAGAGACAACGCCAAGAACACGCTGTATCTCCAATTGAATAGCCTAAAAACTGATGACACGGCCATGTATTACTGTGCAAAAGCAGGTGGTAGTTGGTCACTCAGCGACTGGGGCCAGGGGACCCAGGTCACCGTCTCCTCA |
| Nb30 | CAAGTTCAGTTACAGGAATCTGGGGGAGGCTTGGTGCAGCCTGGGGGGTCTCTGAGACTCTCCTGTGCAGCCTCTGGATTCACCATCAGTAGCTCCAGCATTACCTGGGTCCGCCAGCCTCCAGGGAAGGGACTGGAGTGGGTGTCCAGTCTTTATAGTGTTAGTAGTAACACATTTTATGCAGAGTCCGTGAAGGACCGATTCACCATCTCCGGAGACTACGCCAAGAACACGGTGTATTTGCAAATGAACAGCCTGAAATCTGAGGACACGGCCGTATATTACTGTGCCACTGACCCCCGAATCAGCGTCCCCGATTTGGTGGTAGCTAAAACAATCTCTCGCGCTGACTTTGGCACCTGGGGCCAGGGGACCCAGGTCACCGTCTCCTCA |
| Nb32 | CAGGTGCAGCTGGTGGAGTCTGGGGGAGGCTCGGTGCAGGCTGGAGGGTCTCTGAGACTCTCCTGTACAGCCTCTGGATTCACTTTTGATGATTACGAGATGGGCTGGTACCGCCAGGCTCCAGGGAATGGGTGCGAGTTGGTCTCAACTGTTATTAGTAGTGATGGTAGTACATACTATGTAGACTCCGTGAAGGGCCGATTCACCATCTCCCAAGACAACGCCAAGAACACGGTGTATCTGCAAATGAACAGCCTGAAACCTGAGGACACGGCCGTGTATTACTGTGCGGCCGCGAACGCACTCGTGGGTAACCGAGAATGTGGGCTAGATGACCTTGGTTACTGGGGCCAGGGGACCCAGGTCACCGTCTCCTCA |
| Nb39 | CAGGTGCTGCTGGTGGAGTCTGGGGGAGGCTTGGTGGAGCCTGGGGGGTCTCTCAGACTCTCCTGTTTAGCCTCTGGATTCACCCTCAGTAACTATGCCATGAGCTGGGTCCGCCAGGGTCCAGGGAAGGGACTCGAGTGGGTCTCAACTATTAGCCGTAGTGGTGGTACCCCATACTATACAGACTCCGTGAAGGGCCGATTCACCATCTCCAGAGACAACGCCGGGAACACGCTGTATCTGCAATTGAACAGCCTGAAAACTGAGGACACGGCCATGTATTACTGTACAAGAGGAAACTACGCAGTCGACTATGCCTATGAGTATTACTTTTGGGGCCAGGGAACCCAGGTCACCGTCTCCTCA |
| Nb40 | CAGGTGCAGCTGGTGGAGTCTGGGGGAGGCTCGGTGCAGGCTGGAGGGTCTCTGAGACTCTCCTGTGCAGCCTCTGGATACCCCATCAGTAGCAAGTGCATGGGCTGGTTCCGCCAGGCTCCAGGGGAGGAACGCGAGGGGGTCGCAACTATTTATACCAATGATGGTAGTACATACTATGCCGACTCCGTGAAGGGCCGATTCACCATCTCCCAAGACAACGCCAAGAACACAGTGTATCTGCAAATGAACAGCCTGAAACCTGAGGACACTGCCATGTACTACTGTGCGACAAAGCCCCCCTACTATTATGGTAGCTGCCGTAATCTAAACTCTAGGTATTTTGGTTACTGGGGCCAGGGGACCCAGGTCACCGTCTCCTCA |
| Nb42 | CAGGTGCAGCTGGTGGAGTCTGGGGGAGGCTCGGTGCAGGCTGGAGGGTCTCTGAGACTCTCCTGTGCAGCCTCTGCAGACATCTACAGTTCGAACGTCATGGGCTGGTTCCGCCAGGCTCCAGGGAAGGAGCGCGAGGGGGTCGCAGCTATTTCGCCTGATGGTGGTAACACATACTATGCCGACTCCGTAAAGGGCCGATTCACCATCTCCCGAGACAGCGCCAAGAACACGGTGTATCTGCAAATGAACAGCCTGAAACCTGAGGACACTGCCATGTACTACTGTGCGGCCTCAACCTCCTGGCCTGACTGGGTAGGTGGTCATTTCGCTGACTTGACTTACGTGGCCCAGGGGACCCAGGTCACCGTCTCCTCA |
| Nb44 | CAGGTGCAGCTGGTGGAGTCTGGGGGAGGCTTGGTACAGCCTGGGGGGTCTCTGAGACTCTCCTGTGTAGTTTTTGGATTCACCTTCAGTAGCAGCGGCATGGCCTGGGTCCGCCAGGCTCCAGGGAAGGGGCTGGAGTGGGTGTCCAGTTTGGAGAGTGATGGTACCAACACATTGTATGCAGACTCCGTGAAGGGCCGGTTCACCATCTCCAGAGACAACGCCAAGAACACGGTGTATCTACAAATGAACAGCCTGAAATCTGAGGACACGGCCCTGTATTACTGTCTCACCTCACAGTATGGGCCGAGACACTGGGGCCAGGGGACCCAGGTCACCGTCTCCTCA |
| Nb45 | CAGGTGCAGCTGGTGGAGTCTGGGGGAGGCTTGGTGCAACCTGGGGGGTCTCTGAGACTCTCCTGTGCGGCCTCCGGACCCATCTCCAGTAGTTACCGCGTGAGCTGGGTCCGCCAGGCTCCATGGAAGGGGCTGGAATGGGTGTCCAGTATTCATAGTGATGTAGGTAACACATACTATGCGGGCTCCGTGCTGGGCCGATTCACCATCTCCAGAGACAACGCCAAGAACACGGTGTATCTGCAAATGGACAGCCTGAAATCTGAGGACACGGCCCTGTATTTCTGTGCCGCTGATCCCCGAATCAGTCTCCCCGATTTGCTGGTAGCGCCTACGGTCTCTCTCGCTGACTTTGGTTACTGGGGCCAGGGGACCCAGGTCACCGTCTCCTCA |
| Nb46 | CAGGTGCAGCTGGTGGAGTCTGGGGGAGGCTTGGTGCAGCCTGGGGGGTCTCTGAGACTCTCCTGTGCAGCCTCTGGATTCACCCTCAGTAACTATGTCATGAACTGGGTCCGCCAGGCTCCAGGGAAGGGACTCGAGTGGGTCTCAGCTATTAATAGTGGTGGTAACACATACTATGCAGACTCCGTGAAGGGCCGATTCACCATCTCCAGAGACAACGCCAAGAACACGCTGTATCTGCAATTGAACAGCCTGAAAACTGAGGACGCGGCCATGTATTACTGTGCAAAAGATGGGCCTAAGTACGGTGGTAACTGGTACCTATTTGGTTACTGGGGCCAGGGGACCCAGGTCACCGTCTCCTCA |
| Nb52 | CAGGTGCAGCTGGTGGAGTCTGGGGGAGGCTCGGTGCAGGCTGGAGGGTCTCTGAGACTCTCTTGTACAGCCTCTGAAAACACCTACAGTCGTACCTGCATGGGTTGGTTCCGCCAGGCTCCAGGGAAGGAGCGCGAGGGGGTCGCAACTATTTATACTCGTAGTGGTATGACATACTATGCCGACTCCGTGAAGGGCCGATTCACCATCTCCCAAGACAACAACAAGAACACGTTGTATTTAGAAATGAACACCCTGAAACCTGAAGACACTGCCATGTACTACTGTGCGGCTTCGCCCGTAGGGGACGTCGAATGCGCACTGAAGGAAGGAACCTGGGGCCAGGGGACCCAGGTCACCGTCTCCTCA |
| Nb58 | CAGGTGCAGCTGGTGGAGTCTGGGGGAGGCTCGGTGCAGGCTGGAGGGTCTCTGAGACTCTCCTGTTTAATCTCTGCATACGACTACTTTAAGGCAATGGCCTGGTTCCGCCAGGCTCCAGGGAAGGAGCGCGAGGGGGTCGCTTCTATCTATGGTGGTAACGCATACTATGCGGACTCCGTGCAGGGCCGCGTCACCATCTCCCGAGACAACGCCAAGGCCACGCTGTATCTCCAAATGAACAGCCTGAAACCTGAGGACACTGCCATGTACTACTGTGCGGCCAGTACACGCTATGTACCTACTACTCAGATCCTGCATGAATTTCAATATACCGACTGGGGCCAGGGGACCCAGGTCACCGTCTCCTCA |
| Nb124 | CAAGTACAATTAGTCGAATCAGGCGGTGGCAGCGTCCAGGCTGGCGGGAGCCTCATGCTGTCTTGTGCTGCTTCAGGTTACACAAGTAGCCGGTGCAGTATGGGCTGGTACCGCCAAGCGCCAGGGCTGGAGCGCGAACTCGTCGTAAACATTATCAGCGACGGGTCCATCTGGTACGCGGAGAGCGTAAAAGGTCGGTTTACCGCTTTCCAGGACAATGCCAAGAACATCTTGTACTTACAAATGAACAGTCTCCGCCCGGAGGATACGGCAATGTATTATTGTAATACCGCTACCTGGAACGGTGGGTCGTGTGATAGTGGGGGGCATGGCCGGTACAATTTATGGGGCCAGGGTACGCAAGTCACCGTTAGCAGC |
| Re8-HA1 | ATGGAGAAAATAGTGCTTCTTCTTGCAGTGGTTAGCCTTGTTAAAAGTGATCAGATTTGCATTGGTTACCATGCAAATAACTCGACAGAGCAGGTTGACACGATAATGGAAAAAAACGTCACTGTTACACATGCCCAAGACATACTGGAAAAGACACACAACGGGAGGCTCTGCGATCTGAATGGAGTGAAACCTCTGATTTTAAAGGATTGTAGTGTAGCTGGATGGCTCCTTGGAAACCCAATGTGCGACGAGTTCATCAGAGTGCCGGAATGGTCTTACATAGTGGAGAGGGCTAACCCATCCAATGACCTCTGTTACCCAGGGAACCTCAATGACTATGAAGAACTGAAACACTTATTGAGCAGAATAAACCATTTTGAGAAGACTCTGATCATCCCCAAGAGTTCTTGGCCCGATCATGATACATCATTAGGGGTGAGCGCAGCATGTCCATACCAGGGAATGCCCTCCTTTTTCAGAAATGTGGTATGGCTTATCAAGAAGAACGATACATACCCAACAATAAAGATGAGCTACAATAATACCAATAGGGAAGATCTTTTGATACTGTGGGGGATTCATCATTCCAACAACGCAGCAGAGCAGACAAATCTCTATAAAAACCCAACCACCTATGTTTCCGTTGGGACATCAACATTAAACCAGAGATTGGTGCCCAAAATAGCTACTAGATCCCAAGTAAACGGGCAACGTGGAAGAATGGATTTCTTCTGGACAATTTTAAAACCGAATGATGCAATCCACTTCGAGAGTAATGGAAATTTTATTGCTCCAGAGTATGCATACAAAATTGTCAAGAAAGGGGACTCAACAATCATGAAAAGTGAAATGGAATATGGCCACTGCAACACCAAATGTCAAACTCCAATAGGGGCGATAAACTCTAGTATGCCATTCCACAATATACACCCTCTCACCATCGGGGAATGCCCCAAATACGTGAAATCAAACAAATTAGTCCTTGCGACTGGGCTCAGAAATAATCCTCTAAGAGAGAGGAGAAGAAAAAGAGGACTATTTGGAGCTATAGCAGGGTTTATAGAGGGAGGATGGCAAGGAATGGTAGATGGTTGGTATGGGTACCACCATAGCAATGAACAGGGGAGTGGGTACGCTGCAGACAAAGAATCCACCCAAAAGGCAATAGATGGAGTTACCAATAAGGTCAACTCGATCATTGACAAGATGAACACTCAATTTGAGGCCGTTGGAAGGGAATTTAATAACTTAGAACGGAGAATAGAGAATTTAAATAAGAAAATGGAAGACGGATTCCTAGATGTCTGGACTTATAATGCTGAACTTCTAGTTCTCATGGAAAATGAGAGAACTCTAGATTTCCATGACTCAAATGTCAAGAACCTTTACGACAAAGTCCGACTACAGCTTAGGGATAATGCAAAGGAGCTGGGTAATGGTTGTTTCGAGTTCTATCACAAATGTGATAATGAATGTATGGAAAGTGTAAGAAATGGGACGTATGACTACCCTCAGTATTCAGAAGAAGCAAGATTAAAAAGAGAAGAAATAAGCGGAGTGAAATTGGAATCAATAGGAACTTACCAAATACTGTCAATTTATTCAACAGTGGCGAGTTCCCTAGCACTGGCAATCATTGTGGCTGGTCTATCTTTATGGATGTGCTCCAATGGGTCGTTACAATGCAGAATTTGCATTTAA |

**Table S2. The Characterization of the neutralization concentration.**

| **virus** | **Nanobody** | **HI-IC_50_(μg/mL)** | **MN-IC_50_(μg/mL)** |
| --- | --- | --- | --- |
| Re8/PR8 | Nb10 | 0.10±0.00 | 0.02±0.00 |
| Re8/PR8 | Nb20 | 0.98±0.00 | 0.16±0.03 |
| Re8/PR8 | Nb30 | 4.17±1.04 | 0.05±0.01 |
| Re8/PR8 | Nb42 | 3.26±0.65 | 0.78±0.00 |
| Re8/PR8 | Nb45 | 2.08±0.52 | 0.13±0.03 |
| Re8/PR8 | Nb52 | 7.29±1.82 | 0.91±0.18 |
| Re8/PR8 | Nb58 | >50 | >50 |

**Table S3. Recombinant virus strains used in this study.**

| **Strain** | **HA donor virus(clade)** | **Clade** | | **Source** |
| --- | --- | --- | --- | --- |
| Re6/PR8 | A/duck/Guangdong/S1322/2010 | 2.3.2.1b | This study | |
| Re8/PR8 | A/chicken/Guizhou/4/2013 | 2.3.4.4e | This study | |
| Re10/PR8 | A/duck/Anhui/S1246/2014 | 2.3.2.1e | This study | |
| Re11/PR8 | A/duck/Guizhou/S4184/2017 | 2.3.4.4d | This study | |
| Re12/PR8 | A/chicken/Liaoning/SD007/2017 | 2.3.2.1d | This study | |
| Re14/PR8 | A/whooper swan/Shanxi/4-1/2020 | 2.3.4.4b | This study | |

**Table S4. Recombinant proteins used in this study.**

| **Protein** | **HA1 donor virus(clade)** | **Accession number** | Clade | **Source** |
| --- | --- | --- | --- | --- |
| Re6-HA1 | A/duck/Guangdong/S1322/2010 | EPI869674 | 2.3.2.1b | This study |
| Re8-HA1 | A/chicken/Guizhou/4/2013 | EPI675769 | 2.3.4.4e | This study |
| Re10-HA1 | A/duck/Anhui/S1246/2014 | / | 2.3.2.1e | This study |
| Re11-HA1 | A/duck/Guizhou/S4184/2017 | / | 2.3.4.4d | This study |
| Re12-HA1 | A/chicken/Liaoning/SD007/2017 | / | 2.3.2.1d | This study |
| Re14-HA1 | A/whooper swan/Shanxi/4-1/2020 | EPI1921590 | 2.3.4.4b | This study |

**Table S5. Primers used in this study.**

| **Primer** | | **sequences (5′–3′)** |
| --- | --- | --- |
| Re8-HA1bac-F | | GAAGCGCGCGGAATTCAAAGGATGGTAAGCGCTATTGTTTTATATGTGCTTTTGGCGGCGGCGGCGCATTCTGCCTTTGCGGCGGATCAGATTTGCATTGGTTAC |
| Re8-HA1bac-R | | CTAGTGAGCTCGTCGACGTAGGTTAGTGATGGTGATGATGATGATGATGTCTTTTTCTTCTCCTCTCTCTTAG |
| pFastBac-Dual-F | | CCTACGTCGACGAGCTCACTAG |
| pFastBac-Dual-R | | CCTTTGAATTCCGCGCGCTTC |
| CALL001 | | GTCCTGGCTGCTCTTCTACAAGG |
| CALL002 | | GGTACGTGCTGTTGAACTGTTCC |
| VHH-Forward | | TTCCACCCAAGCAGTGGTATCAACGCAGAGTGGGAGTCTGGRGGAGG |
| VHH-Reverse | | GTATCGATGCCCACCCTCTAGAGGCCGAGGCGGCCGACATGGAGACGGTGACCWGGGT |
| pGADT7-Rec-F | | ATGTCGGCCGCCTCGGCCTCTAGAGGGTGGGCATCGATAC |
| pGADT7-Rec-R | | CCACTCTGCGTTGATACCACTGCTTGGGTGGAA |
| GAL4AD-F | | TACCACTACAATGGATG |
| 3AD-R | | AGATGGTGCACGATGCACAG |
| bait-HA1-F | | CTGCATATGGCCATGGAGGCCGAATTCGATCAGATTTGCATTGGTTAC |
| bait-HA1-R | | ATGCGGCCGCTGCAGGTCGACGGATCCTTATCTTTTTCTTCTCCTCTCTCTTAGAGG |
|  | pGBKT7 -F | GGATCCGTCGACCTGCAGCGGCCGCAT |
|  | pGBKT7 -R | GAATTCGGCCTCCATGGCCATATGCAG |
|  | 5α-Factor-F | TACTATTGCCAGCATTGCTGC |
|  | 3AOX1-R | GGCAAATGGCATTCTGACAT |
|  | P1-F | CCCAAGCTTATGGATCAGATTTGCATTGGTTAC |
|  | P1-R | CTAGCTAGCTCTTTTTCTTCTCCTCTCTCTTAGAGGATTATTTC |
|  | P2-F | CCCAAGCTTATGGATCAGATTTGCATTGGTTAC |
|  | P2-R | CTAGCTAGCACAGAGGTCATTGGATGGGTTAG |
|  | P3-F | CCCAAGCTTATGATAGTGGAGAGGGCTAACCCATC |
|  | P3-R | CTAGCTAGCATTGGTATTATTGTAGCTCATCTTTATTG |
|  | P4-F | CCCAAGCTTATGTACCCAACAATAAAGATGAGCTAC |
|  | P4-R | CTAGCTAGCATTTCCATTACTCTCGAAGTGGATTG |
|  | P5-F | CCCAAGCTTATGCCGAATGATGCAATCCACTTCGAG |
|  | P5-R- | CTAGCTAGCTCTTTTTCTTCTCCTCTCTCTTAGAGGATTATTTC |
|  | P6-F | CCCAAGCTTATGACTCTGATCATCCCCAAGAGTTC |
|  | P6-R | CTAGCTAGCAGCAATAAAATTTCCATTACTCTCG |
|  | P7-F | CCCAAGCTTATGAGGCTCTGCGATCTGAATGG |
|  | P7-R | CTAGCTAGCTTCACTTTTCATGATTGTTGAGTCC |

In primers, K = G or T, W = A or T, R = A or G, Y = C or T, and M = A or C.

The underlined region is the restriction site.

**Table S6. Plasmid used in this study.**

| **Plasmid** | **Relevant characteristic(s)** | **Source** |
| --- | --- | --- |
| pDZ | reverse-genetics vector, Amp^R^ | Laboratory collection |
| pFastBac Dual | Expression vector, Gm^R^, Amp^R^ | Thermo |
| pPICZαA | Expression vector, Zeo^R^ | Invitrogen |
| pGADT7-Rec | Expression vector, Kan^R^ | Clontech |
| pGBKT7 | Expression vector, Amp^R^ | Clontech |
| pYD1 | Expression vector, Amp^R^ | Invitrogen |
| pGBKT7-p53 | Positive control vector, Kan^R^ | Clontech |
| pGADT7-T | Positive control vector, Amp^R^ | Clontech |
| pGBKT7-Lam | Negative control vector, Kan^R^ | Clontech |

**Table S7. Bacterial and yeast strains used in this study.**

|  | **Strain** | **Relevant characteristic(s)** | **Source** |
| --- | --- | --- | --- |
|  | ***E. coli* Strains** | |  |
|  | DH5α | deoR endA1 gyrA96 hsdR17 (rk-mk+) recA1 relA1 supE44 thi-1 Δ(lacZYA-argF) U169 Φ80lacZ ΔM15F - λ - | Vazyme |
|  | DH10Bac | F-, mcrA ∆(mrr-hsdRMS-mcrBC) ϕ80lacZ∆M15 ∆lacX74 recA1 endA1 araD139 ∆ (ara, leu)7697 galU galK λ- rpsL nupG /pMON14272 / pMON7124, Tet^R^ Kan^R^ | Coolaber |
|  | ***Pichia Pastoris* strains** | | |
|  | X33 | Wild-type strain | Laboratory collection |
| ***Saccharomyces cerevisiae* strains** | | | |
|  | Y187 | MATα, ura3-52, his3-200, ade 2-101, trp 1-901, leu 2-3, 112, gal4Δ, met-, gal80Δ, URA3::GAL1UAS-GAL1TATA-lacZ, MEL1 | Coolaber |
|  | Y2HGold | MATa, trp1-901, leu2-3, 112, ura3-52, his3-200, gal4Δ, gal80Δ, LYS2::GAL1UAS-Gal1TATA-His3, GAL2UAS-Gal2TATA-Ade2 URA3::MEL1UAS-Mel1TATA AUR1-C MEL1 | Coolaber |
|  | EBY100 | MATa ura 3-52 trp 1 leu2Δ1 his3Δ200 pep4: HIS3 prb1Δ1.6R can1 GAL | Coolaber |

**Table S8. Interface identification Re8-HA1:Nb10 by PISA Program.**

| **Interface** | | | | | | |
| --- | --- | --- | --- | --- | --- | --- |
| **HA1** | residues | ASA (Å^2^)^a^ | BSA (percentage)^b^ | ΔiG | |  |
|  | TYR 98 | 4.37 | 0.62 || | 0.1 01 | |  |
|  | ASP 131 | 29.56 | 17.57 |||||| | -0.26 | |  |
|  | SER 133 | 83.54 | 8.71 || | -0.1 | |  |
|  | LEU 133a | 115.74 | 112.40 |||||||||| | 1.41 | |  |
|  | GLY 134 | 9.19 | 1.68 || | 0.03 | |  |
|  | VAL 135 | 70.04 | 47.86 ||||||| | 0.32 | |  |
|  | SER 136 | 7.13 | 6.71 |||||||||| | 0.1 | |  |
|  | ALA 137 | 72.45 | 10.40 || | 0.05 | |  |
|  | PRO 145 | 74.59 | 19.36 ||| | 0.31 | |  |
|  | TRP 153 | 19.56 | 16.09 ||||||||| | 0.26 | |  |
|  | ILE 155 | 19.24 | 19.08 |||||||||| | 0.31 | |  |
|  | LYS 156 | 45.9 | 40.92 ||||||||| | -0.08 | |  |
|  | LYS 157 | 83.36 | 19.24 ||| | 0.31 | |  |
|  | ASN 158 | 132.77 | 34.67 ||| | -0.14 | |  |
|  | ASP 159 | 75.25 | 48.70 ||||||| | -0.1 | |  |
|  | ALA 188 | 70.31 | 15.18 ||| | 0.23 | |  |
|  | ALA 189 | 61.44 | 48.75 |||||||| | 0.74 | |  |
|  | GLU 190 | 42 | 11.23 ||| | -0.1 | |  |
|  | THR 192 | 50.35 | 21.00 ||||| | 0.18 | |  |
|  | ASN 193 | 96.41 | 87.12 |||||||||| | -0.59 | |  |
|  | LEU 194 | 36.82 | 36.32 |||||||||| | 0.58 | |  |
|  | GLN 226 | 37.67 | 7.75 ||| | -0.13 | |  |
| **Nb10** | | | | |  | |
|  | ARG 29 | 70.35 | 7.86 || | -0.09 | |  |
|  | ASP 49 | 0.12 | 0.12 ||||||||||| | 0.23 | |  |
|  | ASN 52 | 83.1 | 75.06 |||||||||| | -0.03 | |  |
|  | GLY 53 | 45.96 | 25.99 |||||| | -0.03 | |  |
|  | ASN 54 | 65.91 | 65.76 |||||||||| | -0.64 | |  |
|  | THR 55 | 56.49 | 41.52 |||||||| | -0.37 | |  |
|  | TYR 56 | 27.31 | 27.31 ||||||||||| | 0.42 | |  |
|  | TYR 57 | 77.89 | 26.87 |||| | -0.23 | |  |
|  | PRO 58 | 70.12 | 14.54 ||| | -0.13 | |  |
|  | THR 59 | 82.18 | 3.10 | | 0.05 | |  |
|  | VAL 60 | 159.25 | 71.11 ||||| | 1.14 | |  |
|  | PRO 98 | 44.92 | 8.53 || | 0.14 | |  |
|  | LEU 99 | 9.89 | 6.88 ||||||| | -0.08 | |  |
|  | TRP 100 | 142.3 | 56.46 |||| | 0.9 | |  |
|  | PRO 101 | 58.17 | 53.02 |||||||||| | 0.56 | |  |
|  | THR 102 | 128.1 | 120.47 |||||||||| | 0.94 | |  |
|  | LEU 103 | 119.64 | 72.29 ||||||| | 1.16 | |  |
|  | GLN 106 | 19.2 | 10.49 |||||| | -0.12 | |  |
|  | PHE 107 | 45.1 | 2.19 | | 0.04 | |  |

^a^ BSA: Buried Surface Area; ^b^ ||||: Buried area percentage, one bar per 10%.

**Table S9. The confidence and binding bonds of HA1: nanobody protein complex.**

| Complex | ipTM/ pTM | N_HB_ | N_SB_ |
| --- | --- | --- | --- |
| Re8-HA1: Nb10 | 0.90/0.82 | 14 | 0 |
| Re8-HA1: Nb20 | 0.85/0.81 | 13 | 0 |
| Re8-HA1: Nb30 | 0.88/0.82 | 14 | 0 |
| Re8-HA1: Nb42 | 0.88/0.82 | 10 | 0 |
| Re8-HA1: Nb45 | 0.74/0.76 | 13 | 0 |
| Re8-HA1: Nb52 | 0.14/0.63 | 7 | 5 |
| Re14-HA1: Nb58 | 0.27/0.66 | 12 | 6 |

A pTM score exceeding 0.5 indicates that the predicted fold of the complex may resemble the actual structure. ipTM assesses the precision of the predicted relative positions of the subunits inside the complex. Values beyond 0.8 indicate confident, high-quality forecasts, whilst values below 0.6 imply a probable failure in prediction. ipTM scores ranging from 0.6 to 0.8 represent an ambiguous area where predictions may be accurate or inaccurate. N_HB_, Hydrogen bond. N_SB_, Salt bridge.

**Table S10. Sequence conservation of nanobodies epitopes in all H5.**

| **Position^a^** | **Nb10** | **Nb20** | **Nb30** | **Nb42** | **Nb45** | **Nb52** | **Consensus^b^ (%)** |
| --- | --- | --- | --- | --- | --- | --- | --- |
| 77 |  |  |  |  |  | D | D (99.1) |
| 80 |  |  |  |  |  | I | I (68.6), L (26.1), T (4.0) |
| 98 | Y | Y |  |  | Y |  | Y (100) |
| 125a |  |  |  |  |  | K | K (75.7), R (22.5) |
| 131 | D | D | D | D | D | D | E (73.5), D (17.2) |
| 132 |  |  |  |  |  | T | T (49.3), A (45.8), S (4.6) |
| 133 | S |  |  | S | S | S | S (97.9) |
| 133a | L | L | L | L | L | L | L (58.4), S (35.0) |
| 134 | G | G | G | G | G | G | G (99.8) |
| 135 | V | V | V | V | V | V | V (98.2) |
| 136 | S | S | S | S | S |  | S (99.2) |
| 137 | A | A | A | A | A | A | A (62.4), S (36.2) |
| 141 |  |  |  |  |  | Y | Y (96.1), H (1.1) |
| 142 |  |  |  |  |  | Q | Q (73.8), N (15.5), L (5.5) |
| 143 |  |  |  | G |  | G | G (98.2) |
| 144 |  | M | M | M | M | M | A (34.9), R (24.2), N (9.4), T (8.9), V (7.4), K (6.0), S (3.7), M (3.3) |
| 145 | P | P | P | P | P | P | P (52.1), S (42.3) |
| 146 |  |  |  |  |  | S | S (99.6) |
| 147 |  |  |  |  |  | F | F (99.7) |
| 149 |  |  |  |  |  | R | R (99.1) |
| 153 | W | W | W | W | W |  | W (100) |
| 155 | I | I | I | I | I | I | I (83.9), T (13.1) |
| 156 | K | K | K | K | K |  | K (98.3) |
| 157 | K | K | K | K | K | K | K (99.1) |
| 158 | N | N | N | N | N |  | N (87.7), D (10.1) |
| 159 | D | D | D | D | D |  | D (55.2), N (34.3), S (8.6) |
| 186 |  | N | N |  | N |  | N (98.2) |
| 187 |  | N | N |  | N |  | N (48.6), D (44.8), S (5.3) |
| 188 | A | A | A |  | A |  | A (84.2), E (13.2) |
| 189 | A | A | A | A | A |  | A (54.8), E (40.2) |
| 190 | E | E | E | E | E |  | E (98.6) |
| 192 | T | T | T |  | T |  | T (89.4), I (6.4), A (1.7) |
| 193 | N | N | N | N | N |  | N (46.4), K (28.8), R (16.5) |
| 194 | L | L | L | L | L | L | L (98.1), I (1.7) |
| 222 |  |  |  | Q | Q |  | Q (52.2), K (45.1), R (1.5) |
| 225 |  | G |  | G | G |  | G (99.8) |
| 226 | Q | Q | Q | Q | Q |  | Q (99.0) |
| 227 |  | R | R | R | R |  | S (51.1), R (45.2), Q (1.3) |
| 228 |  | G |  |  | G |  | G (99.9) |
| Av^c^(%) | 80.9 | 76.9 | 74.1 | 75.2/ | 76.8 | 77.6 |  |
| Av^d^(%) | 83.5 | 80.4 | 78.0 | 79.1 | 80.0 | 81.8 |  |

^a^ Residue at position based on the numbering of HA from H3 viruses.

^b^ Total 3000 sequences available for H5 viruses in the NCBI Influenza Database and GISAID EpiFlu Database at the time of download on November, 2024.

^c^ Average conservation of most common residue in H5 subtype.

^d^ Average conservation of the residue of Re8-HA.

**Table S11. Interaction analysis of Re8-HA1:Nb10 by PISA Program.**

| **Interaction** | | | |
| --- | --- | --- | --- |
| **Types^c^** | **HA1** | **Distance. [Å]** | **Nb10** |
| H | ASN 158[N] | 2.71 | ASN  52[O] |
| H | ASP 159[N] | 3.44 | GLY  53[O] |
| H | ASP 159[N] | 3.04 | ASN  54[OD1] |
| H | ASN 158[N] | 3.35 | ASN  54[OD1] |
| H | LYS 156[NZ] | 2.62 | THR  55[O] |
| H | ASN 193[ND2] | 2.84 | TYR  57[O] |
| H | VAL 135[N] | 3.44 | THR 102[OG1] |
| H | ASP 131[OD2] | 3.22 | ASN  52[ND2] |
| H | LYS 156[O] | 2.74 | ASN  54[ ND2] |
| H | ASP 159[OD1] | 3.25 | THR  55[N] |
| H | ASP 159[OD2] | 2.80 | THR  55[OG1] |
| H | ASN 193[OD1] | 2.95 | TYR  57[N] |
| H | VAL 135[O] | 2.74 | THR 102[OG1] |
| H | LEU 133a[O] | 2.83 | THR 102[OG1] |

^a^ H, Hydrogen bond.

**Table S12. The comparison of antigenic epitopes.**

| **Position^a^** | **Nb10** | **FLD21.140** | **AVFluIgG03** |
| --- | --- | --- | --- |
| 98 | Y |  |  |
| 131 | D | E | E |
| 133 | S | S | S |
| 133a | L | L |  |
| 134 | G |  | G |
| 135 | V | V | V |
| 136 | S | S | S |
| 137 | A | S | S |
| 143 |  | R |  |
| 144 |  | K | T |
| 145 | P | S | P |
| 153 | W | W | W |
| 155 | I | I | I |
| 156 | K | K | K |
| 157 | K |  | K |
| 158 | N | N | N |
| 159 | D | S | N |
| 188 | A |  |  |
| 189 | A | A |  |
| 190 | E | E |  |
| 192 | T |  |  |
| 193 | N | K | K |
| 194 | L | L | L |
| 222 |  |  | K |
| 225 |  |  | G |
| 226 | Q |  | Q |
| Counts^b^ | 22 | 18 | 19 |

^a^ Residue at position based on the numbering of HA from H3 viruses.

^b^ The number of antigenic amino acids recognized by the antibody.

**Table S13. The comparison of residues between the interacted sites of Re8 and Re14 recognized by Nb10 and the download sequences.**

| **Position** | **A/chicken/Guizhou/4/2013（Re8）** | **A/whooper swan/Shanxi 4-1/2020（Re14）** | **A/dairy cow/USA/24 024344-001/2024** | **A/dairy cow/USA/24 011574-001/2024** | **A/dairy cow/Texas/24 015973-027/2024** | **A/dairy cow/Colorado/24 025575-001/2024** | **A/dairy cow/USA/24 029900-001/2024** | **A/dairy cow/Colorado/24 024946-001/2024** | **A/dairy cow/USA/24 027472-001/2024** | **A/dairy cow/Idaho/BI-ME-015/2024** |
| --- | --- | --- | --- | --- | --- | --- | --- | --- | --- | --- |
| 98 | Y | Y | Y | Y | Y | Y | Y | Y | Y | Y |
| 131 | D | E | E | E | E | E | E | E | E | E |
| 133 | S | S | S | S | S | S | S | S | S | S |
| 133a | L | L | L | L | L | L | L | L | L | L |
| 134 | G | G | G | G | G | G | G | G | G | G |
| 135 | V | V | V | V | V | V | V | V | V | V |
| 136 | S | S | S | S | S | S | S | S | S | S |
| 137 | A | A | A | A | A | A | A | A | A | A |
| 145 | P | P | P | P | P | P | P | P | P | P |
| 153 | W | W | W | W | W | W | W | W | W | W |
| 155 | I | I | I | I | I | I | I | I | I | I |
| 156 | K | K | K | K | K | K | K | K | K | K |
| 157 | K | K | K | K | K | K | K | K | K | K |
| 158 | N | N | N | N | N | N | N | N | N | N |
| 159 | D | D | D | D | D | D | D | D | D | D |
| 188 | A | A | A | A | A | A | A | A | A | A |
| 189 | A | E | E | E | E | E | E | E | E | E |
| 190 | E | E | E | E | E | E | E | E | E | E |
| 192 | T | I | T | T | T | T | T | T | T | T |
| 193 | N | N | N | N | N | N | N | N | N | N |
| 194 | L | L | L | L | L | L | L | L | L | L |
| 222 | Q | Q | Q | Q | Q | Q | Q | Q | Q | Q |

**Fig. S1 Presentation of the selecting procedure and nanobody expression.**


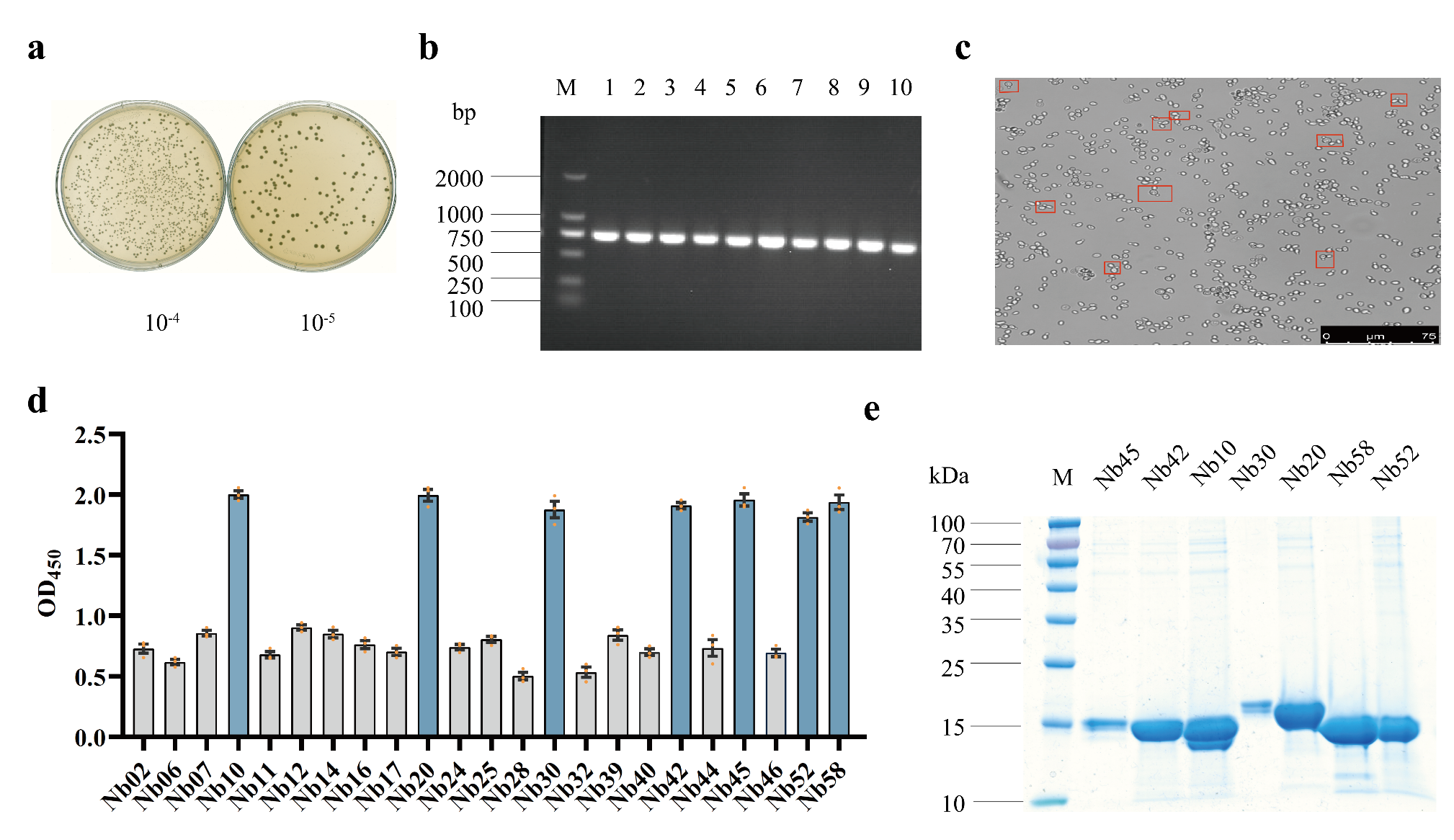


**Fig. S1 Presentation of the selecting procedure and nanobody expression.** a. Quantification of the yeast two-hybrid (Y2H) nanobody library. A 10 μL library aliquot that had been diluted 100,000 times was spread out on SD/-Leu plates, and an average of 150 colonies were grown on the plate. This experiment was carried out in duplicated. Therefore, the titer of the Y2H library was about (1.5 ± 0.06) × 10^9^ cfu/mL. SD/-Leu, synthetic dropout medium lacking Leucine. b. The correct insertion rate of the Y2H library. 10 single colonies picked randomly were identified through PCR analysis to assess the insertion rate of the library. The size of the PCR fragments from all individual colonies is approximately 750 bp. M denoted the DL2000 DNA marker. c. Presentation of the mating procedure. The mated cells were inspected for the presence of zygotes under microscope. A zygote commonly exhibits a three-lobed structure or a shape similar to a “mickey mouse”. Some zygotes were marked with red boxes. The more zygotes there are, the higher mating efficiency is, which means the higher probability of screening for interacting nanobodies. d. Binding capacity of selected nanobodies. After being confirmed the interaction was genuine, the obtained positive clones were sequenced. The VHHs genes were inserted into pPICZα and transformed into X33 for production and purification. The identified nanobodies were further confirmed for binding potency by indirect ELISA. Seven of the twenty-three nanobodies that were separated had high absorbance values, which means these nanobodies showed significant interaction with the antigen. Data were expressed as mean ± SEM (n = 3). e. SDS-PAGE analysis purity and molecular size of nanobody with his tag. The protein was isolated by 15% gel under reducing conditions with the prestained protein marker displayed on the left side of the gel image. The nanobodies expressed by X33 with a hexahistidine tag at the C-terminal are around 15 kDa, and the purity is higher than 95%.

**Fig. S2 Binding characteristics of neutralizing nanobodies.**

**
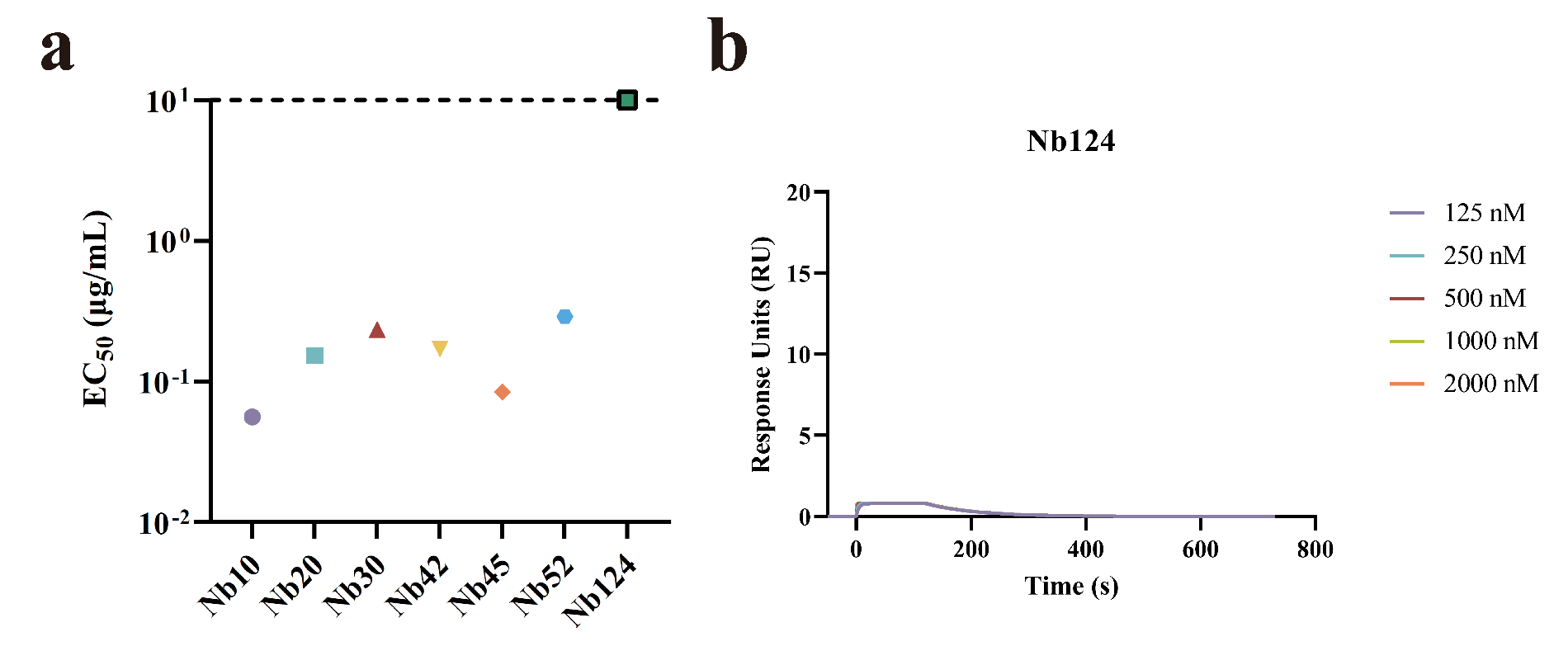
**

**Fig. S2 Binding characteristics of neutralizing nanobodies.** a. Summary of half-maximal effective concentrations (EC_50_) against Re8-HA1. Six nanobodies with relatively higher neutralizing activities were selected to determine the binding ability of nanobodies to HA1. Nb124 were used as controls. The dashed line indicated 10 μg/mL. b. The curves of responses and kinetic of Nb124 and Re8-HA1. The Nb124 varying from 2000 to 125 nM were exposed to immobilized HA1, and a response signal was detected. With a low response, Nb124 served as a negative control.

**Fig. S3 SDS-PAGE analysis of expression of the HA1s.**

**
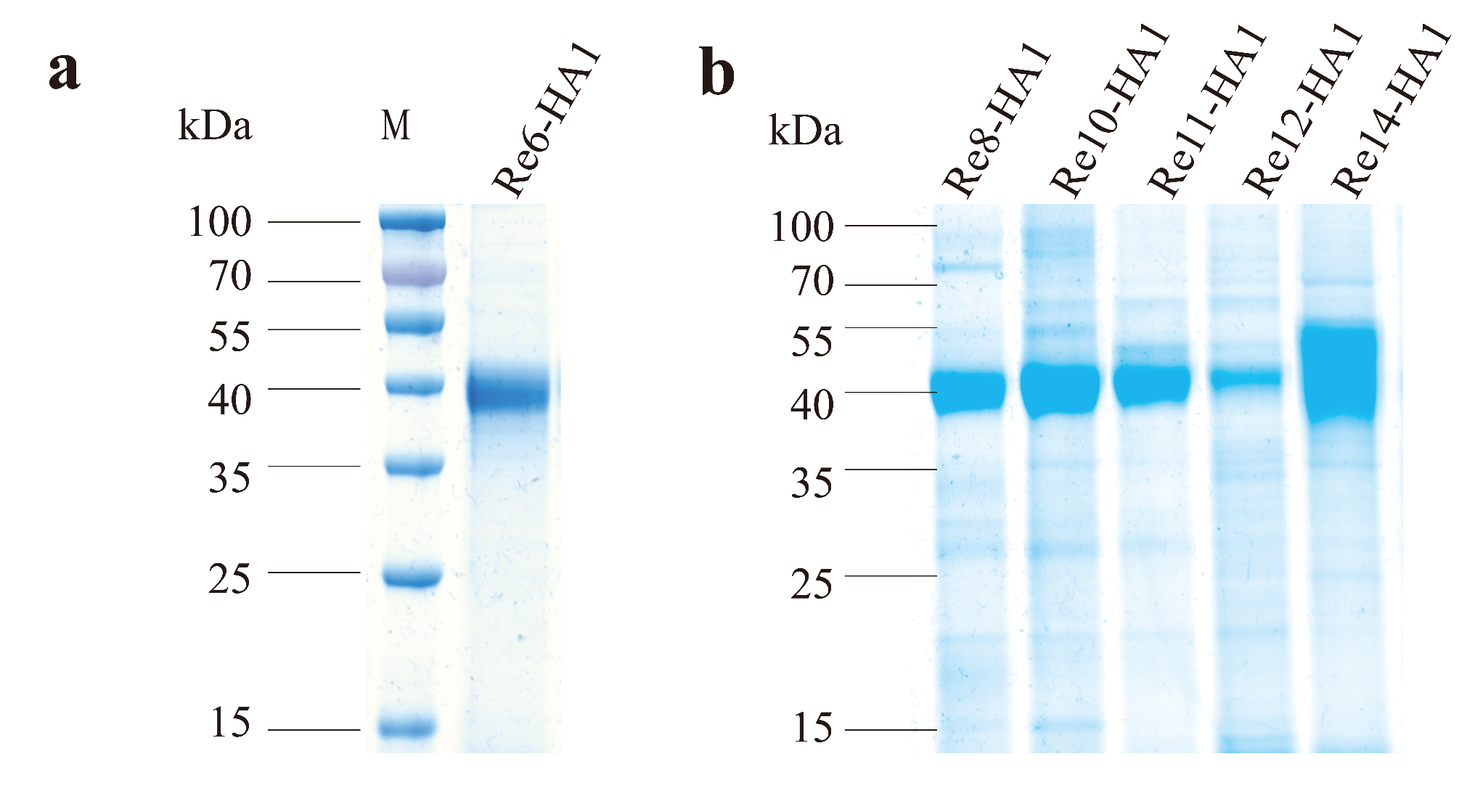
**

**Fig. S3 SDS-PAGE analysis of expression of the HA1s.** A and B. The baculovirus expressed HA1s as C-terminal his tag proteins. The purity of these HA1s was analyzed by SDS-PAGE and was more than 95%. The 12% gel shown has a band at approximately 40 KDa, respectively.

**Fig. S4** **Structures analysis of nanobodies separately binding to HA1 by AlphaFold 3.**

**
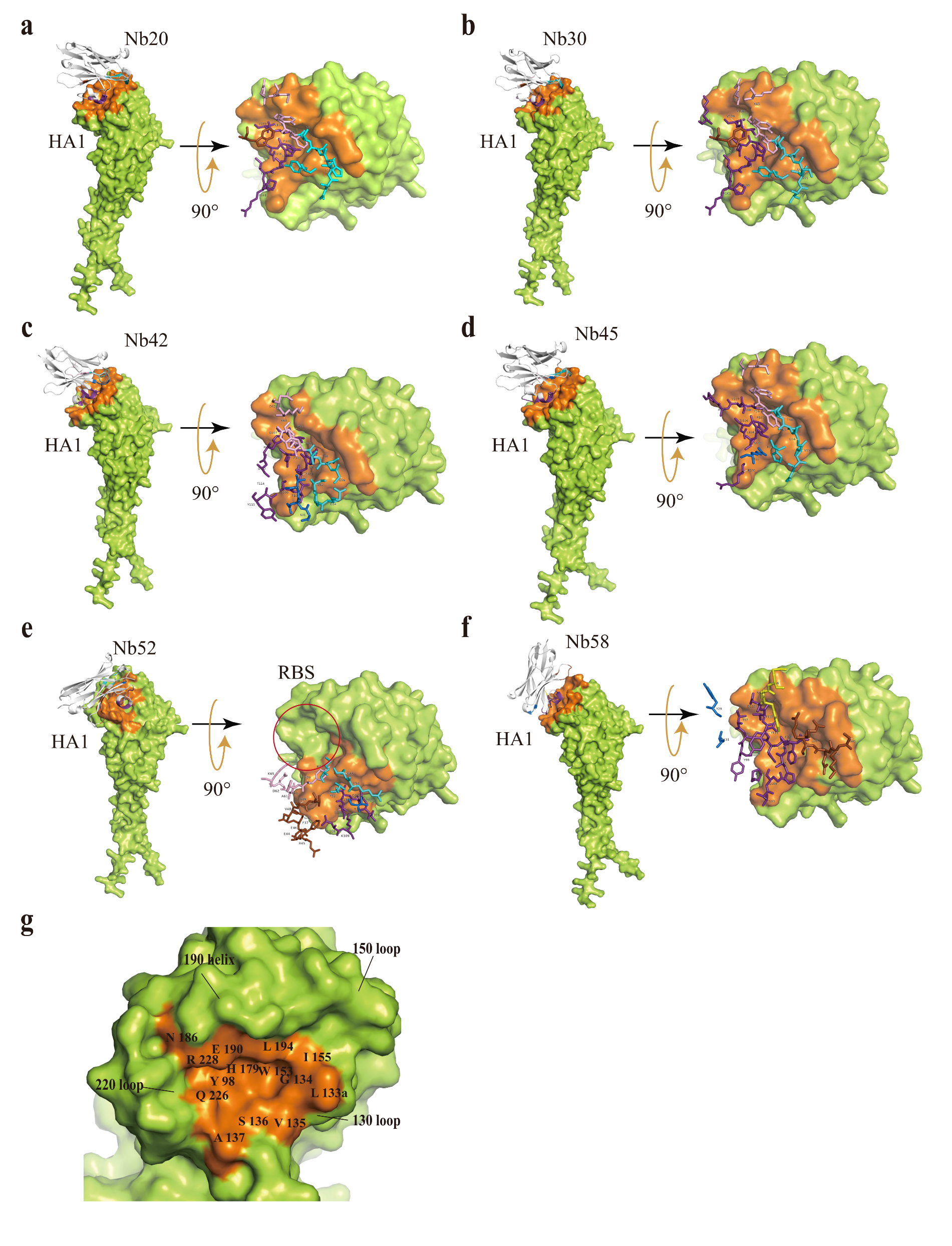
**

**Fig. S4 Structures analysis of nanobodies separately binding to HA1 by AlphaFold 3.** a–f. All CDRs and FRs involved binding of nanobodies were labeled and marked with different colors. The stick represented CDR1 in marine, FR2 in brown, CDR2 in cyan, FR3 in light pink, CDR3 in violet-purple and FR4 in yellow. All HA1s were represented in surface with limon, while the orange denoted the binding sites. The panels a–e represented the structure analysis of Nb20, Nb30, Nb42, Nb45 and Nb52 binding to Re8-HA1. the panel f indicated the structure analysis of Nb58 binding to Re14-HA1. The results showed that the motifs Y98, S133, L133a, V135, S136, A137, G143, A144, P145, W153, I155, K156, K157, N158, D159, N186, N187, E189, E190, I192, N193, L194, K196, Q222, G225, Q226, R227, and G228 of Re14-HA1 were involved in binding. g. The RBS region of H5 influenza virus subtype HAs. The HA1 was represented in surface with limon, while the orange denoted the RBS of the Re8-HA1.
